# New Quinoid Bio-Inspired Materials Using Para-Azaquinodimethane Moiety

**DOI:** 10.3390/molecules29010186

**Published:** 2023-12-28

**Authors:** Walaa Zwaihed, François Maurel, Marwan Kobeissi, Bruno Schmaltz

**Affiliations:** 1 Laboratoire de Physico-Chimie des Matériaux et des Electrolytes Pour l’Energie (PCM2E)EA6299, Université de Tours, 37200 Tours, France; walaa.zwaihed@etu.univ-tours.fr; 2 Laboratoire Rammal Rammal, Equipe de Synthèse Organique Appliquée SOA, Faculté des Sciences 5, Université Libanaise, Boulevard Nabih Berri, Quartier des Universités, Nabatieh 6573/14, Lebanon; mkobeissi@ul.edu.lb; 3 Université Paris Cité, CNRS, ITODYS, 75013 Paris, France; maurel@u-paris.fr

**Keywords:** para-azaquinodimethane (p-AQM), quinoid, Knoevenagel condensation, bio-inspired materials, bandgap engineering

## Abstract

Quinoid single molecules are regarded as promising materials for electronic applications due to their tunable chemical structure-driven properties. A series of three single bio-inspired quinoid materials containing para-azaquinodimethane (p-AQM) moiety were designed, synthesized and characterized. **AQM1**, **AQM2** and **AQM3,** prepared using aldehydes derived from almonds, corncobs and cinnamon, respectively, were studied as promising quinoid materials for optoelectronic applications. The significance of facile synthetic procedures is highlighted through a straightforward two-step synthesis, using Knoevenagel condensation. The synthesized molecules showed molar extinction coefficients of 22,000, 32,000 and 61,000 L mol^−1^ cm^−1^, respectively, for **AQM1**, **AQM2** and **AQM3**. The HOMO-LUMO energy gaps were calculated experimentally, theoretically showing the same trends: **AQM3** < **AQM2** < **AQM1**. The role of the aryl substituent was studied and showed an impact on the electronic properties. DFT calculations show planar structures with quinoidal bond length alternation, in agreement with the experimental results. Finally, these bio-based materials showed high thermal stabilities between 290 °C and 340 °C and a glassy behavior after the first heating–cooling scan. These results highlight these bio-based single molecules as potential candidates for electronic or biomedical applications.

## 1. Introduction

Organic electronic technologies have attracted tremendous attention since the beginning of the 21st century. Their flexibility, lightweight, their interesting optical and electronic properties and their ability to tune these properties depending on structural chemistry modifications, organic semiconductors (OSCs) show high advantages for modern technologies [1]. Organic photovoltaics (OPV) [2,3] and light emitting diodes (OLED) [4,5,6] are used for renewable energy, and organic field effect transistors (OFET) [7,8] are used for electronic paper or the Internet of things. However, long multi-step synthetic routes and utilization of non-environment friendly solvents or organometallic catalysts for coupling reactions are still an issue [9,10]. It is necessary to consider these parameters for the future commercialization of the greenish generation of OSCs. In order to introduce the green chemistry principles in organic synthesis, the use of natural products, atom economy or minimal waste production should be achieved [11]. In fact, several studies using green synthetic strategies to prepare OSC have already been proposed, like Knoevenagel condensation [12,13,14].

Quinoidal molecules are a subclass of OSCs known for efficient π-electron delocalization, near-infrared absorption, and narrow band gap due to the minimization of the bond length alternation [15]. A large variety of quinoidal structures was intensively studied for both n-type and p-type semiconductors due to their high planarity, efficient charge transport and delocalization of π electrons [16]. Thus, structures including benzodipyrrolidone (BDP) [17], naphthodipyrrolidone (NDP) [18], benzo[1,2-b:4,5-b’]-dithiophene-2,6-dione (BDTD) [19] and quinodimethanes (QDM) moieties have been developed. Among them, QDMs have attracted attention to rationalize the origin of tuning between their diradical and quinoidal character. Research on the potential applications for these compounds has also extended to hybrid supramolecular structures like polybromide salts containing quinolinium-type cations for redox flow batteries [20,21]. In a different approach, tetracyanoquinodimethane derivatives have been employed to afford high conductivity used as dopants in organic semiconductors [22]. However, the high reactivity of QDMs has hindered their integration in conjugated semiconducting systems [23]. In 2017, an ambient stable quinoid unit (para-azaquinodimethane: p-AQM) was first introduced by Liu and co-workers [24]. This latter moiety contains nitrogen atoms in the central 6-member ring and alkoxy substituents on the periphery, which bring additional solubility and stability. The combination of facile synthetic accessibility, high chemical stability, low optical band gaps and good electronic properties makes p-AQM a promising candidate in low band gap polymers for organic photovoltaics, OFETs and thermoelectric devices [24,25,26,27]. The introduction of AQM moiety into conjugated polymers leads to a unique quinoid modulation of electronic structure and chain conformation unlike conventional donor−acceptor polymers [28]. Nevertheless, the application of p-AQM as a single molecule is rather modest. Wang et al. proposed a new singlet fission donor–acceptor chromophore within the p-AQM skeleton and bearing bis-thiophene groups on both sides [29]. Unless there is excellent stability after 30 days, AQTT exhibits strong visible light absorption and suitable triplet energy of 1.1 eV leading to a 165% triplet yield [29]. More recently, a smart study of p-AQM-based fluorophores was published by M. R. Rao et al. [30]. By selecting the side groups, the single molecules exhibit good fluorescence quantum yields. Halochromic properties were also explored with trifluoracetic acid and fluorescence quenching of 90% with donor–acceptor Q5.

**Figure 1:**
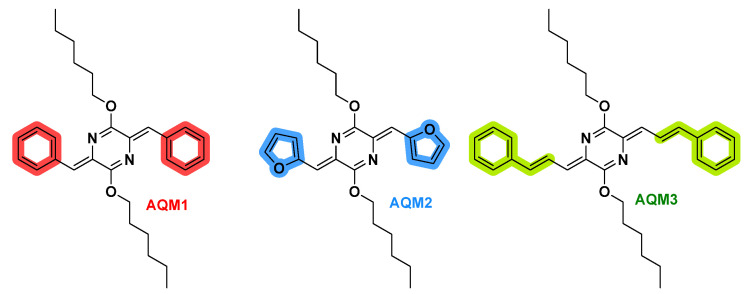
Structure of the quinoidal p-AQM-based molecules (**AQM1**, **AQM2** and **AQM3**).

**Scheme 1:**
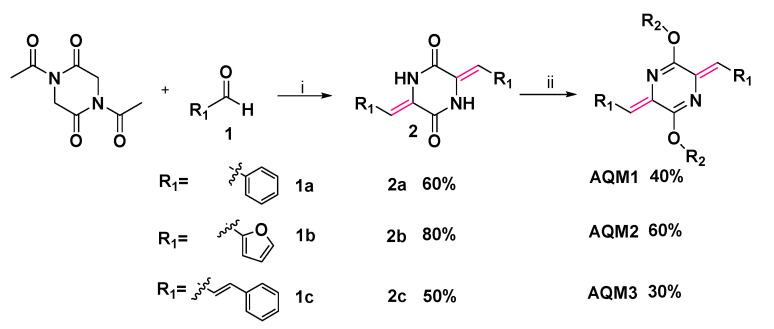
Synthetic route of target p-AQM molecules (**AQM1–3**), i: NEt_3_, DMF, 120 °C, 24 h, ii: Br-C_6_H_13_, K_2_CO_3_, DMF, 100 °C, 2 h, R_2_=C_6_H_13_.

Here, we report the synthesis of three new p-AQM-based quinoidal molecules derived from natural aldehyde precursors (Figure 1). The synthetic strategy is based on two easy synthetic steps (Scheme 1), using metal-free and non-halogenated solvent reaction (Knoevenagel condensation), as a key step. The optical, electronic and thermal properties were studied depending on the aryl side substituents. In order to study this structure–property relationship, benzyl, furanyl and vinylbenzyl moieties are selected as substituents grafted on both sides of the quinoidal p-AQM core. By increasing the conjugation length or the aromaticity of the aryl substituents, the HOMO/LUMO energy gap, the molar extinction coefficient or the thermal behavior can be tuned in agreement with theoretical studies. This work helps to develop new bio-inspired quinoidal building blocks with suitable properties as a promising class of electronic materials.

## 2. Results and Discussion

### 2.1. Synthesis 

The preparation of the p-AQM single molecules was successfully accomplished following a two-step synthetic route based on modified literature procedures [24,26,31] (Scheme 1). The first step is a Knoevenagel condensation between commercially available N,N’-diacetylglycine anhydride and three natural aldehyde precursors. Although the three aldehyde precursors used in this study are commercially available chemicals, they can be directly extracted from natural origins, and so, highlight the integration of nature-inspired aldehydes in the synthetic route of these materials. For example, Benzaldehyde (**1a**) could be extracted from bitter almond oil [32], furaldehyde (**1b**) is a product of dehydration of sugars from corncobs, oats and wheat bran [33] and trans cinnamaldehyde (**1c**) could be obtained from the bark of cinnamon tree [34]. After in situ cleavage of the acetyl groups, the intermediates **2a**, **2b** and **2c** were obtained in good yields, respectively, 60, 80 and 50% yields. As described in the literature, compounds **2a–c** adopt a Z,Z’-configuration due to N-O acetyl transfer [35]. The subsequent alkylation under mildly basic conditions (K_2_CO_3_) leads to the formation of target quinoid p-AQM derivate molecules in 40, 60 and 30% yields for **AQM1**, **AQM2** and **AQM3,** respectively. Attempts to optimize the reaction yields by changing the base (NaH or Cs_2_CO_3_), reaction times (24 h) or temperature (120 °C) were unsuccessful and resulted in the formation of N-alkylated untargeted products. The competition between N and O-alkylation is attributed to the stabilization of the negative charge formed on N or O depending on the nature of the involved base (Hard Soft Acid Base theory) [36]. Considering the design of the quinoidal molecules, alkoxy groups play a double role in stabilizing and solubilizing the quinoidal planar structures. The addition of hexyl chains on both sides of the p-AQM moiety allows high enough solubility in various protic and aprotic solvents such as CHCl_3_, CH_2_Cl_2_, toluene, hexane or THF. The solubility issue is of importance in organic optoelectronic applications and more particularly in the deposition of semiconductor materials by solution-processed methods.

### 2.2. Optical and Electrochemical Properties

**Figure 2:**
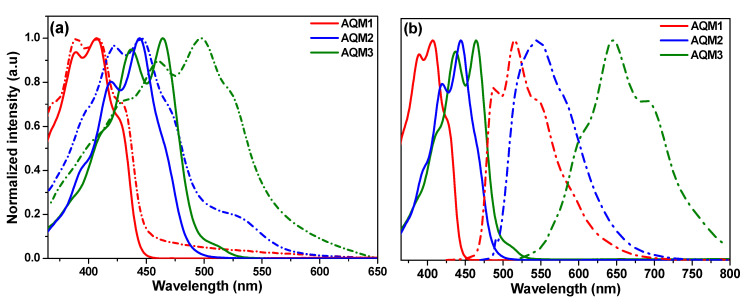
(**a**) Normalized UV-visible absorption spectra in 10^−5^ M chloroform solution (full line) and in films on glass substrate (short dash-dotted line). (**b**) Absorption and fluorescence spectra (dash-dotted line) in 10^−5^ M chloroform solution.

In order to study their optical and electronic properties, the absorption spectra of the three p-AQM molecules were performed in chloroform solution and in thin films (Figure 2a). The photoluminescence (PL) spectra were performed in chloroform (Figure 2b). The pertinent data are summarized in Table 1. The quinoidal compounds are colored (Figure S1a) and absorb strongly in the visible region with absorption maxima between 407 to 464 nm in solution. These bands could be attributed to the strongly optically allowed S_0_-S_1_ electronic transition [29], with a vibronic fine structure. Compared to the previous non-quinoidal intermediates **2a–c** (Figure S3), a redshift of 60–80 nm was observed and is directly related to the increase in π-conjugation and formation of the quinoidal structure. The aryl substitution in **AQM1-3** has an impact on the absorption maxima. A maximum at 407 nm was observed for the benzyl group (**AQM1**) which is approximately the same for tolyl moiety in **Q1** [30]. By substitution to furan moiety, a 37 nm redshift is clearly observed. The reason could be the decrease in the ring strain and degree of aromaticity from benzene to furan which lowers the electron delocalization energy, thus contributing to better conjugation and molecular coplanarity [37]. The redshift is even more pronounced by the addition of a vinyl linker from **AQM1** to **AQM3**. A 57 nm redshift is shown and could be explained by the π-extended conjugation leading to a significant bathochromic effect on the absorption maxima. The effect of solvent polarity on the absorption spectra was also studied (Figure S2, Table S1). Although a shift in polar solvents compared to non-polar solvents was observed, the shift was shown to be weak, especially for **AQM1** and **AQM2** with a maximum shift of 2–5 nm. However, a slightly larger shift of 9 nm was detected for **AQM3** when increasing solvent polarity from hexane to DMF (extended conjugation) [38]. The same trend has already been observed in the literature for tolyl, anisyl and naphthyl grafted moieties [30]. The molar extinction coefficients (ε) of the p-AQM molecules were measured at maximum absorption (Figures S4–S6). **AQM3** shows the highest extinction coefficient around 61,000 L mol^−1^ cm^−1^. For example, such a strong absorption coefficient is quite favourable for singlet fission materials [29]. **AQM1** and **AQM2** showed lower ε_max_, respectively, around 22,000 and 32,000 L mol^−1^ cm^−1^. The molar extinction coefficient of **AQM3** was almost three times that of **AQM1**. Nevertheless, it was much lower than 6H-[1,2,5]thiadiazolo[3,4-e]thieno[3,2-b]indole flanked para-azaquinodimethane reported in the literature by Yu et al. (170,000 L mol^−1^ cm^−1^) [39]. The optical band gaps were calculated from the onset of absorption at a maximum wavelength in solution. The molecules showed a decrease in the band gap upon increasing conjugation following the corresponding order: **AQM1** (2.75 eV) > **AQM2** (2.55 eV) > **AQM3** (2.50 eV). Values are in good agreement with those found in the literature. Liu et al. [24] reported a thiophene-flanked AQM monomer (**AQM-ref**) with a band gap of 2.43 eV. More recently, Rao et al. synthesized an aryl-flanked AQM series showing band gaps between 2.3 to 2.7 eV, in agreement with our study [30]. In the solid state, the three molecules did not have the same trend. **AQM1** and **AQM2** showed little difference from the dilute chloroform solution, with broad absorption peaks. However, **AQM3** revealed a broader absorption spectrum with a maximum absorption shift from 464 nm to 497 nm (33 nm), which could be due to π–π interactions [40].

Photoluminescence (PL) measurements were also performed on the p-AQM single molecules in chloroform solution (Figure 2b, Table 1). The compounds **AQM1-3** emit in chloroform solution, with emission colors varying from light blue to dark orange (Figure S1b), and emission maxima between 515–650 nm. The three molecules exhibit a single emission band with a vibronic fine structure for **AQM1** and **AQM3**. Regarding the substitution effect, the emission maxima followed a similar trend on the redshift pattern as the absorption spectra: benzyl (**AQM1**, 515 nm) < furanyl (**AQM2**, 545 nm) < vinylbenzyl (**AQM3**, 645 nm). The Stokes shift was calculated and the compounds showed larger Stokes shifts compared to the literature. While in aryl and thiophenyl substituted AQM [30], the Stokes shift is approximately 80 nm, **AQM1** and **AQM2** showed Stokes shifts around 100 nm and **AQM3** of 180 nm. The fluorescence quantum yields (ϕ_f_) of the three target molecules, measured in chloroform solution, show values between 0.20 and 0.48 (Table 1). These results are in agreement with the literature showing, for small molecules, ϕ_f_ in the range of 0.2 to 0.58 [29,30].

**Figure 3:**
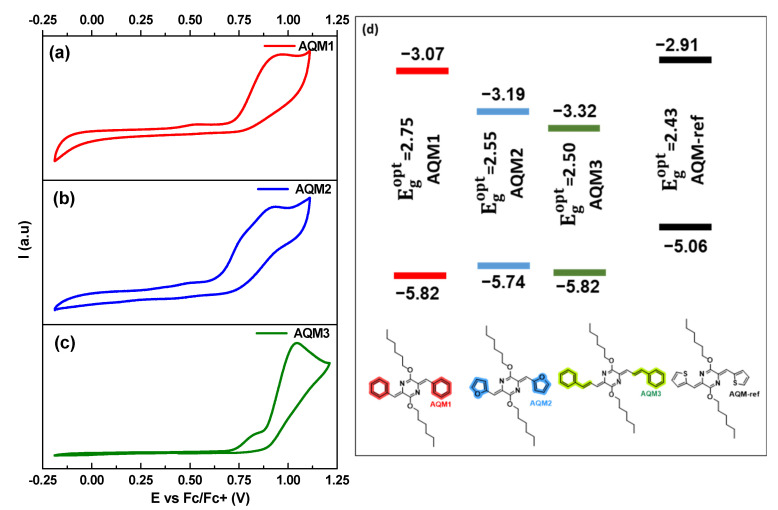
(**a**–**c**) Cyclic voltammograms of p-AQM molecules in 10^−2^ M CH_2_Cl_2_ solution containing 0.1 M tetrabutylammonium perchlorate as supporting electrolyte at 50 mV/s scan rate. (**d**) Comparison of frontier energy levels and band gaps of target molecules compared to the reported **AQM-ref** [24].

To investigate the energy levels of p-AQM-based molecules, cyclic voltammetry (CV) measurements were performed in degassed CH_2_Cl_2_ solution, using a conventional three-electrodes setup and the ferrocene/ferrocenium (Fc/Fc^+^) redox couple as an internal reference. Figure 3a–c shows cyclic voltammograms of the three molecules and the data are summarized in Table 1. On the anodic sweeps, the three molecules show irreversible oxidation waves. HOMO energy levels were estimated from the onset of the first oxidation peak and were found to be similar for **AQM1** and **AQM3** with a value of −5.82 eV, while **AQM2** shows a slightly higher HOMO level of −5.74 eV. This effect has already been reported in the literature showing that benzyl moiety is more aromatic than furanyl moiety leading to a stabilization of the HOMO level [41]. The obtained HOMO values are within the reported range of donor–flanked p-AQMs, such as thiophene-substituted **AQM-ref** [24] (Figure 3d). LUMO energy levels were further calculated from HOMO levels and $E_{g}^{opt}$ in solution and found to be −3.07 eV, −3.19 eV and −3.32 eV, respectively, for **AQM1**, **AQM2** and **AQM3**. Clearly, the effect of the substituents (from benzyl to furanyl and vinylbenzyl group) on the p-AQM is more pronounced on the LUMO level than HOMO level, with **AQM3** having the deepest LUMO probably due to extended conjugation [42]. 

**Table 1:** A summary of the relevant data obtained by optical, electrochemical and computational characterizations of the synthesized p-AQM molecules.

	Absorption				Emission		CV		DFT			
	$\lambda_{max}^{sol}$/nm	${E_{opt}^{g}}^{a}$/eV	ε ^b^ /M^−1^ cm^−1^	$\lambda_{max}^{film}$ /nm	λ_em_ ^c^ (ϕ_f_) ^d^ /nm	Δλ ^e^ /nm	E_HOMO_^f^/eV	E_LUMO_^g^/ eV	λ_max_ /nm	E_HOMO_/eV	E_LUMO_ /eV	$E^{g}$ /eV
AQM1	407	2.75	2.2 × 10^4^	408	515 (0.48)	108	−5.82	−3.07	400	−5.646	−2.382	3.263
AQM2	444	2.55	3.2 × 10^4^	445	545 (0.2)	101	−5.74	−3.19	434	−5.319	−2.344	2.975
AQM3	464	2.50	6.1 × 10^4^	497	645 (0.32)	181	−5.82	−3.32	465	−5.305	−2.574	2.734

^a^ optical band gap calculated from onset of absorption in solution according to the following equation: $E_{opt}^{g\;}\;$ **=** $\frac{1240}{\lambda onset}$, ^b^ extinction coefficient, see supporting information for calculation details, ^c^ maximum emission wavelength, ^d^ fluorescence quantum yield measured using fluorescein as reference (ϕ_f_ = 0.925 in 0.1 M NaOH [43])**,** ^e^ Stokes shift, ^f^ HOMO energy level calculated using equation $E_{HOMO}\;$= −e($E_{ox}+5.1\;eV)$, ^g^ LUMO energy level calculated using equation $E_{LUMO}$ = $E_{HOMO}$ + $E_{g}^{opt}\;(solution)$

### 2.3. Thermal Properties

The thermal properties of the p-AQM-based synthesized molecules were determined by thermo-gravimetric analysis (TGA) and differential scanning calorimetry (DSC) measurements. The TGA and DSC plots are depicted in Figure S7 whereas Table S2 summarizes the relevant data of both measurements. All molecules show relatively high thermal stability with decomposition temperatures (Td) of 340, 312 and 292 °C, respectively, for **AQM1**, **AQM2** and **AQM3**. These materials can be deposited in thin film and are stable to commonly used thermal annealing conditions [44]. DSC plots show thermal transition for all the molecules between 40–115 °C. Melting endothermic peaks (Tm) were confirmed by the capillary method (Table S2). After the first cycle, the p-AQM derivative molecules seem to stay in an amorphous structure but any glass transition temperature could be observed from the 2nd heating cycle.

### 2.4. Theoretical Calculations

**Figure 4:**
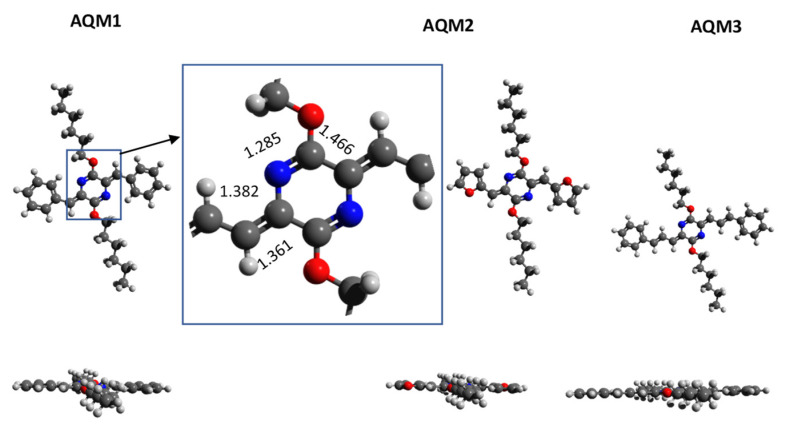
Optimized geometries of the most stable isomers of p-AQM molecules (top view and side view). PBE0/6-311G(d,p) level of calculation.

In order to better understand the electronic, optical and redox properties of p-AQM molecules, extensive density functional theory (DFT) and time-dependent DFT (TD-DFT) calculations were performed using Gaussian 16. In this section, we summarize the main obtained results, and the reader is referred to the Supplementary Material for further details, particularly those relating to the properties of the different calculated isomers or redox potentials (the standard hydrogen electrode potential, V_SHE_, was taken as 4.43 eV [45]). In order to test the accuracy of the DFT and TD-DFT calculations to reproduce ground and excited states properties, benchmark calculations were performed with several exchange-correlation functionals (see Supplementary Materials for details). PBE0 functional in combination with the 6–311G(d,p) basis set was selected to optimize the ground state geometry of the three compounds. Considering the calculations of the excited state, ωB97XD functional was selected for its accuracy with the experimental data. All the calculations were performed taking into consideration chloroform as the solvent. The geometries and energies of different possible isomers for each studied compound were determined at different levels of calculations (see Supplementary Materials for details). In this study, theoretical conformation Z/E (symmetrical conformations on both sides of the AQM moiety) were taken on bonds c,d and e (Figure S8). Among the possible isomers (2 for **AQM1**, 4 for **AQM2** and 8 for **AQM3**), most stable isomers were demonstrated. Considering c bond on the three compounds, Z conformation is the most stable, which is in agreement with the literature [24,35]. Z, ZE and ZEE, respectively, for **AQM1**, **AQM2** and **AQM3** are the most stable isomers and show high planarity derived from the conjugated structure with dihedral angles Φ_1_, Φ_2_ and Φ_3_ at 180° (Table S4). Contrary to thiophene-based AQM where S-N interactions force the molecule to be ZZ configuration (**AQM-ref**) [24], there are no interactions between N-O in **AQM2**, containing furanyl groups. In addition, the bond length calculations of the central ring show a strong quinoidal character with a significant difference between the length of C=N double bonds (very short bond a length of 1.285 Å) and the length of single C-N bonds (length b of 1.382 Å) (Figure 4). The bond length alternation (BLA) was calculated on the red dot line pathway (Figure S8) as the difference between the average of the single and the double bonds in the conjugated path. As the degree of π-electron delocalization increases, the length of the single (double) bonds along the path will decrease (increase), and consequently, the BLA value will become smaller. Therefore, a smaller magnitude of BLA implies better electron conjugation along the selected path. The calculated BLA are found to be 0.110, 0.096 and 0.095 Å, respectively, for **AQM1**, **AQM2** and **AQM3**, showing a stronger quinoidal character for **AQM1**. Interestingly, the BLA value corresponds more or less to the difference between the single C-N and double C=N bond length in the AQM unit. It could be concluded that the BLA of the synthesized compounds and isomers is largely dominated by the geometry of the AQM unit.

**Figure 5:**
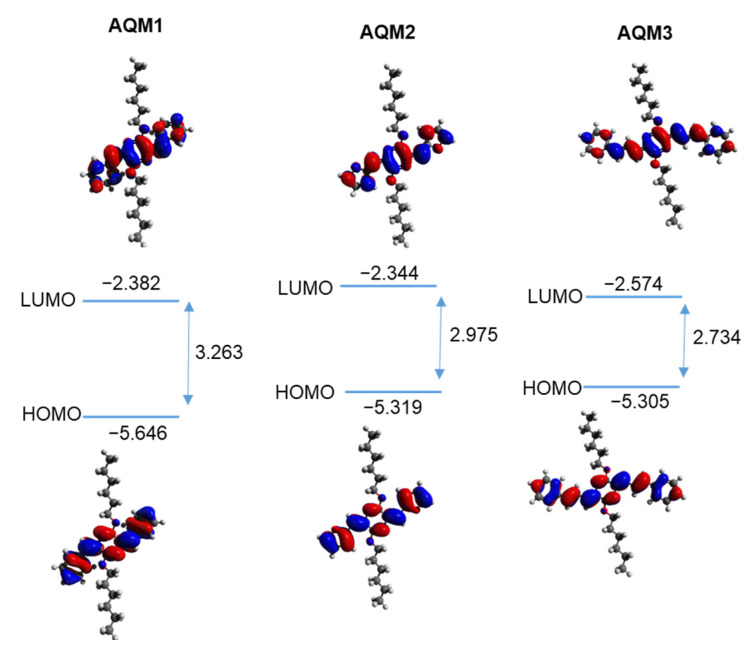
Calculated molecular orbital distribution and energy levels of p-AQM molecules (HOMO, LUMO and HOMO-LUMO gap in eV, PBE0/6-311G(d,p) calculations.

DFT calculations were also used to study the molecular orbital distribution and understand the trend in HOMO-LUMO energy gaps. The calculated energies of the HOMO are found −5.646 eV, −5.319 eV and −5.305 eV, respectively, for **AQM1**, **AQM2** and **AQM3** and are in relatively good agreement with experimental values (−5.82 eV, −5.74 eV, −5.82 eV, respectively). As shown in Figure 5, the molecules show a similar HOMO-LUMO energy gap trend compared to that deduced from optical absorption in the following order: **AQM3** (2.734 eV) < **AQM2** (2.975 eV) < **AQM1** (3.263 eV). Noticeably, the distribution of the HOMO and LUMO of the three molecules are distributed on the whole π conjugated system, i.e., not only the AQM core but also the conjugated substituent. In addition, TD-DFT calculations were also performed (Figure S11). The results obtained with ωB97XD functional (see supporting information for a benchmark study) demonstrate a relatively good agreement between experimental and theoretical values of maximum absorption wavelengths.

The calculated S_0_ → S_1_ vertical electronic transition of the studied compounds is listed in Table S6 and compared with the experimental values. The calculated spectra for the lowest energy isomers are presented in Figure S11. The calculations give accurate results with an error of less than 15 nm (λcalc: 465 and 464 nm for **AQM3**, λcalc: 400 and 407 nm for **AQM1** and λcalc: 434 and 444 nm for **AQM2**). For each compound, the S_0_ → S_1_ vertical transition corresponds to a HOMO → LUMO excitation.

Emission spectra calculated predict a large Stokes shift in agreement with experimental findings: 81 nm for **AQM1**, 78 nm for **AQM2** and 161 nm for **AQM3** (see Table S8). Remarkably, the compound with the most extensive conjugated path, i.e., **AQM3**, offers the largest Stokes shift. This can be explained by a greater geometric relaxation in the excited state.

## 3. Materials and Methods

### 3.1. Chemicals and Reagents 

All reactions were carried out in oven-dried glassware sealed with rubber septa under an inert atmosphere and were stirred using Teflon-coated magnetic stir bars. Triethylamine was distilled before use, and dry DMF over molecular sieves from Fisher Scientific was used for all reactions. All commercially available chemicals and solvents (dry and argon bubbled chloroform and dichloromethane solutions were used for UV and CV) were purchased from Sigma Aldrich, TCI Europe, Alfa Aesar, Acros Organics and Ficher Scientific and were used without further purification. Deuterated solvents were purchased from Eurisotop and used as received. All reactions were monitored by thin-layer chromatography (TLC) carried out on 0.25-mm silica gel plates (60 F-254) using UV light (254 nm, 365 nm) for visualization. Silica gel (60 A, 40–63 μ) from Carlo Erba was used for column chromatography. 

### 3.2. Characterisation

NMR spectra were recorded on a Bruker Avance 300 (300 MHz) spectrometer unless otherwise indicated. Chemical shifts (δ) are reported in parts per million (ppm) and all coupling constants (J) are expressed in Hertz (Hz). Mass spectra were recorded on a Finnigan MAT 8500 using an ionization energy of 70 eV (electron). UV-Vis spectra were recorded with a Jasco V-670 spectrometer. The emission spectra were recorded with fluoromax-4 from Horiba. The excitation wavelength was fixed at 407 nm, 445 nm and 465 nm, respectively, for **AQM1**, **AQM2** and **AQM3**. The measurements were conducted in 10^−5^ M CHCl_3_ solution. Fluorescence quantum yields of **AQM1-3** were calculated in chloroform solution at different concentrations, taking fluorescein in 0.1 M NaOH as standard reference [43]. A common excitation wavelength for the sample and reference was selected to record the emission spectra of each molecule (438 nm, 452 nm and 475 nm for **AQM1**, **AQM2** and **AQM3,** respectively). Cyclic voltammetry (CV) was performed on a Biologic Applied Research MPG2 multi-channel potentiostat, and CV experiments were performed at room temperature with a conventional three-electrode setup consisting of a platinum disk working electrode, silver wire and platinum wire, respectively, as reference and counter electrodes. The potential of the reference electrode was calibrated using Fc/Fc^+^ couple as an internal standard. All the measurements were conducted in anhydrous dichloromethane media (10^−2^ M concentration) under a nitrogen atmosphere using Bu_4_NClO_4_ (0.1 M) as a supporting electrolyte at a scan rate of 50 mV/s. Differential scanning calorimetry (DSC) measurements were performed on Perkin-Elmer DSC-400 (heating/cooling rate 10 °C/·min). Thermogravimetric analysis (TGA) was fulfilled using a Perkin Elmer STA 6000 at a heating rate of 10 °C/·min under N_2_. Melting point determination by the capillary method was performed by Stuart Scientific SMP3 Melting Point Apparatus. 

### 3.3. Experimental Procedures 

#### 3.3.1. Synthesis of Intermediates **2a**, **2b** and **2c** by Knoevenagel Condensation

Into a mixture of 1,4-Diacetyl-2,5-piperazinedione (1 eq, 5 mmol) and aromatic aldehyde (2.3 eq, 11.6 mmol), in DMF (24 mL) was syringe injected triethylamine (4 eq, 20 mmol) at 120 °C under argon. Upon addition, the original colorless solution turned dark orange to brown. A precipitate was formed during the overnight reaction. The reactions were stopped after 24 h, cooled to room temperature and placed in an ice bath for 1 h. The precipitate formed was collected by filtration and rinsed with water, ethyl acetate and methanol. The solids obtained were pure enough for the next step.

**2a** was obtained as yellow solid (0.87 g, 60% yield). (^1^H NMR, DMSO *d*_6_, 300 MHz): δ = 10.30 (s, 2H), 7.55 (d, *J* = 7.3 Hz, 4H), 7.42 (t, *J* = 7.4 Hz, 4H), 7.33 (t, *J* = 6.7 Hz, 2H), 6.78 (s, 2H). (^13^C NMR, DMSO *d*_6_, 75 MHz): δ = 158.02, 133.12, 129.32, 128.68, 128.16, 126.45, 114.95, 39.52. HRMS: M + 1 found 291.11, theoretical: 291.32. The analytic data are in accordance with the literature [26].

**2b** was obtained as light brown solid (1 g, 80% yield). (^1^H NMR, DMSO *d*_6_, 300 MHz): δ = 9.52 (s, 2H), 7.93 (d, *J* = 1.8 Hz, 2H), 6.91 (d, *J* = 3.5 Hz, 2H), 6.69 (s, 2H), 6.67 (dd, *J* = 3.5, 1.8 Hz, 2H). (^13^C NMR, DMSO *d*_6_, 75 MHz): δ = 156.25, 149.87, 145.01, 123.47, 114.64, 112.46, 101.94, 54.86, 39.52. HRMS: M + 1 found 271.07, theoretical: 271.24. The analytic data are in accordance with the literature [31].

**2c** was obtained as light orange solid (0.85 g, 50% yield). (^1^H NMR, DMSO *d*_6_, 300 MHz): δ = 10.81 (s, 2H), 7.71–7.63 (m, 2H), 7.60 (d, *J* = 7.2 Hz, 4H), 7.39 (t, *J* = 7.3 Hz, 4H), 7.31 (d, *J* = 6 Hz, 2H), 6.99 (d, *J* = 15.3 Hz, 2H), 6.74 (d, *J* = 12.1 Hz, 2H). (^13^C NMR, DMSO *d*_6_, 75 MHz): δ = 171.99, 163.42, 160.25, 137.78, 136.80, 128.76, 127.01, 126.34, 121.92, 117.99, 46.07, 39.52, 26.80. HRMS: M + 1 found 343.14, theoretical: 343.39.

#### 3.3.2. Synthesis of Target p-AQM Molecules by Alkylation

A mixture of the diarylene-diketopiperazine **2** (1 eq, 1.1 mmol), K_2_CO_3_ (5 eq, 5.5 mmol) and 1-bromohexane (4 eq, 4.4 mmol) in DMF (10 mL) was stirred at 100 °C for 2 h under argon atmosphere. After cooling to room temperature, the reaction mixture was filtered, and the precipitate was washed with ethanol to afford the desired products.

**AQM1** was obtained as a yellow solid (0.2 g, 40% yield). (^1^H NMR, CDCl_3_, 300 MHz): δ = 8.11 (d, *J* = 8.5 Hz, 4H), 7.37 (t, *J* = 7.4 Hz, 4H), 7.30 (s, 2H), 6.87 (s, 2H), 4.41 (t, *J* = 6.7 Hz, 4H), 1.97–1.79 (m, 4H), 1.41 (m, 12H), 0.93 (t, *J* = 7.0 Hz, 6H). (^13^C NMR, CDCl_3_, 75 MHz): δ = 158.42, 131.39, 128.37, 128.16, 122.58, 122.36, 77.16, 67.09, 31.76, 28.79, 27.47, 26.15, 22.81, 14.23. HRMS: M + 1 found 459.30, theoretical: 459.63

**AQM2** was obtained as a dark brown solid (0.28 g, 60% yield). (^1^H NMR, CD_2_Cl_2_, 400 MHz): δ = 7.51 (dd, *J* = 1.7, 0.5 Hz, 2H), 7.31 (d, *J* = 3.5 Hz, 2H), 6.84 (s, 2H), 6.59–6.53 (m, 2H), 4.38 (t, *J* = 6.5 Hz, 4H), 1.93–1.80 (m, 4H), 1.52–1.32 (m, 12H), 0.93 (t, *J* = 7.1 Hz, 6H). (^13^C NMR, CD_2_Cl_2,_ 75 MHz): δ = 158.44, 152.94, 143.35, 129.07, 114.78, 113.23, 111.07, 67.39, 54.56, 54.20, 53.84, 53.84, 53.48, 53.12, 32.02, 29.03, 26.39, 23.04, 14.23. HRMS: M + 1 found 439.26, theoretical: 439.56.

**AQM3** was obtained as a dark orange solid (0.16 g, 30% yield). (^1^H NMR, CD_2_Cl_2_, 300 MHz): δ = 7.79 (dd, *J* = 15.9, 11.4 Hz, 2H), 7.52 (d, *J* = 7.3 Hz, 4H), 7.34 (t, *J* = 7.4 Hz, 4H), 7.25 (t, *J* = 7.2 Hz, 2H), 6.83 (d, *J* = 15.9 Hz, 2H), 6.74 (d, *J* = 11.3 Hz, 2H), 4.39 (t, *J* = 6.6 Hz, 4H), 1.91–1.75 (m, 4H), 1.51–1.34 (m, 12H), 0.93 (t, *J* = 7.0 Hz, 6H). (^13^C NMR, CD_2_Cl_2,_ 75 MHz): δ = 157.43, 138.02, 135.86, 132.02, 129.07, 128.39, 127.20, 124.99, 124.29, 66.72, 54.56, 54.20, 53.84, 53.84, 53.48, 53.12, 32.06, 29.18, 26.35, 23.07, 14.26. HRMS: M + 1 found 511.33, theoretical: 511.71

### 3.4. Computational Details

#### 3.4.1. Ground State Calculation

The molecular calculations of **AQM1**, **AQM2** and **AQM3** were carried out with the Gaussian 16 [46] program package in the frame of density functional theory (DFT). The ground state structures were fully optimized at the DFT level of theory using the PBE0, B3LYP and ωB97X-D exchange-correlation functionals (XCF) with the 6-311G (d,p) basis set. These three XCFs were used for optimization to compare their impact on the geometries. Tight SCF convergence criteria (10^−8^ a.u.) and an integration grid of 10^−8^ were used for all calculations. Frequency calculations were performed on the optimized geometries to verify the nature of the computed geometries and the lack of imaginary values in the wavenumber calculations confirmed the successful optimization as a minimum geometry.

#### 3.4.2. Absorption and Emission Spectra

Ground-state optimized structures were used to compute absorption wavelength maxima (λ_max_) and oscillator strength (f) for the first 10 lowest excited states. Only the S_0_ → S_1_ electronic transition is discussed in the text, as it is the only transition found with significant oscillator strength in the region where the compounds absorb. Molecular excitation energies were calculated using time-dependent density functional theory (TD-DFT). Six different XCFs are tested and their ability to correctly reproduce the lowest energies transition and maximum absorption of the studied compounds are evaluated. We assessed a representative set of exchange-correlation functions, namely, PBE0 [47], B3LYP [48], M06 [49], M06-2X [49], CAM-B3LYP [50] and ωB97X-D [51], which were all combined with the 6-311G(d,p) basis set. To determine the emission energies and similarly to the ground state, the first-singlet-excited-state structures of the studied compounds are fully optimized and characterized at the TDDFT level. Emission energies were computed in the State-Specific (SS) [52] formalisms within the PCM/TD-DFT method.

For both ground state and excited state calculations, solvent effect (Chloroform) was undertaken for ground state optimization and TD-DFT calculations by means of the Polarizable Continuum Model (PCM) using the integral equation formalism (IEFPCM).

## 4. Conclusions

Three new p-AQM stable quinoid molecules based on natural aldehydes were successfully designed, synthesized and characterized. The three molecules display good solubility in common solvents, showing hexyl chains are suitable for solubility issues. The facile synthetic strategy allowed us to obtain the quinoid materials in relatively satisfactory reaction yields. The utilization of three different natural aldehydes as starting materials was helpful in tuning the optoelectronic properties. The role of the end groups flanked by the quinoidal core was studied through experimental measurements and theoretical calculations. Increasing the electron density by introducing five-membered furan rings redshifts the absorption and destabilizes the HOMO level in **AQM2** compared to **AQM1**. Indeed, increasing the conjugation length over the π-backbone showed a significant influence on both the optical and electrochemical properties. **AQM3** showed a bathochromic shift of maximum absorption by 57 nm compared to **AQM1**, and a reduction in optical band gap. The values of the bandgaps are in good agreement with the theoretical calculation, meaning the increase in Eg **AQM3** < **AQM2** < **AQM1**. The synthesized molecules showed molar extinction coefficients of 22,000, 32,000 and 61,000 L mol^−1^ cm^−1^, respectively, for **AQM1**, **AQM2** and **AQM3**. These latter quinoid molecules exhibited high thermal stabilities with decomposition temperatures ranging from 292 to 340 °C. DSC analysis revealed the amorphous behaviour of p-AQM target molecules after the first heating scan. In order to explicate the optimized geometries and molecular orbital distribution, DFT calculations were performed, and they revealed high planarity of p-AQMs and strong quinoidal character through single and double bond length alternation. In conclusion, these bio-inspired quinoid materials, which allow us to tune the electronic properties, could be interesting in the nowadays nature- and technology-oriented society. As perspectives, an integration of these materials in redox flow batteries as ammonium salts or in photovoltaic devices as specific light absorbers can be considered. As nature-inspired materials, their use in medical applications could be of interest too. A study of these quinoid materials in biological activity is currently ongoing. 

## Data Availability

Data are contained within the article and Supplementary Material.

## Supplementary Materials

The following supporting information can be downloaded at: https://www.mdpi.com/article/10.3390/molecules29010186/s1, Figure S1: Photos showing the colors of p-AQM molecules in chloroform solutions (a) and under UV light of 254 nm (b). Figure S2: Normalized UV/visible absorption spectra of **AQM1**, **AQM2** and **AQM3** in different solvents. Table S1: Maximum absorption wavelengths of **AQM 1-3** in different solvents. Figure S3: Comparison of normalized UV/visible absorption spectra of (a) non-quinoidal intermediates **2a-c,** and (b) p-AQM molecules in 10^−5^ M THF solution. Figure S4: (a) UV/visible absorption spectra of **AQM1** at different concentrations in CHCl_3_, (b) Absorbance vs Concentration plot of **AQM1** in CHCl_3_. Figure S5: (a) UV/visible absorption spectra of **AQM2** at different concentrations in CHCl_3_, (b) Absorbance vs Concentration plot of **AQM2** in CHCl_3_. Figure S6: (a) UV/visible absorption spectra of **AQM3** at different concentrations in CHCl_3_, (b) Absorbance vs Concentration plot of **AQM3** in CHCl_3_. Figure S7: (a): TGA curves under nitrogen at 10 °C /min scan rate. (b): DSC curves under nitrogen: first heating (full line) and cooling cycles (dashed line) at 10 °C/min scan rate. Table S2: Thermal properties of p-AQM molecules. Figure S8: E, Z, ZZ, ZE and ZEE isomers displayed for **AQM1-3**. Table S3: Relatives energies (in kJ.mol^−1^) of the isomers of AQM1, AQM2. Figure S9: Optimized geometries of the isomers of **AQM1**, **AQM2** and **AQM3** (ZEE) compounds (PBE0/6-311G(d,p) calculations) (top view and side view) and **AQM3** compounds calculated at the PBE0/6-311G(d,p) and B3LYP/6-311G(d,p) levels. Table S4: Geometric parameters of the isomers of **AQM1**, **AQM2** and **AQM3** compounds calculated at the PBE0/6-311G(d,p) and B3LYP/6-311G(d,p) levels. Table S5: Energies (in eV) of HOMO, LUMO and HOMO/LUMO gap of the isomers of AQM1, AQM2 and AQM3 compounds calculated at the PBE0/6-311G(d,p), B3LYP/6-311G(d,p) and ωB97XD/6-311G(d,p) levels. **Figure S10:** HOMO and LUMO orbitals for **AQM1**, **AQM2** and **AQM3** obtained at the PBE0/6-311G(d,p) level of calculations. Table S6: Benchmark on transitions for **AQM3** (ZEE isomer), **AQM1** (Z isomer) and **AQM2 (ZE isomer)** compounds. The ground state geometries were computed using the 6-311G(d,p) basis-set using PBE0, B3LYP and ωB97XD functionals. Figure S11: TD-DFT calculations of the absorption spectra of **AQM1** (Z), **AQM2** (ZE) and **AQM3** (ZEE). Table S7: Lowest electronic transition energies calculated for **AQM3**, **AQM1** and **AQM2** compounds at the ωB97XD/6-311G(d,p) PBE1PBE/6-311G(d,p). Table S8: Lowest electronic transition energies in absorption and emission calculated for **AQM3** (ZEE isomer), **AQM1** (Z isomer) and **AQM2** (ZE isomer) compounds at the ωB97XD/6-311G(d,p) PBE1PBE/6-311G(d,p) level. Table S9. Computed redox potentials vs. experimental ones of the **AQM1**, **AQM2** and **AQM3** compounds. For each compound, calculation were performed on the most stable isomer at the PBE0/6-311(g,d) level. Figure S12: ^1^ H NMR spectrum of **2a** (DMSO d_6_, 300 MHz). Figure S13: ^13^ C NMR spectrum of **2a** (DMSO d_6_, 75 MHz). Figure S14: ^1^ H NMR spectrum of **2b** (DMSO d_6_, 300 MHz). Figure S15: ^13^ C NMR spectrum of **2b** (DMSO d_6_, 75 MHz). Figure S16: ^1^ H NMR spectrum of **2c** (DMSO d_6_, 300 MHz). Figure S17: ^13^ C NMR spectrum of **2c** (DMSO d_6_, 75 MHz). Figure S18: ^1^H NMR spectrum of **AQM1** (CDCl_3_, 300 MHz). Figure S19: ^13^C NMR spectrum of **AQM1** (CDCl_3_, 75 MHz). Figure S20: ^1^H NMR spectrum of **AQM2** (CD_2_Cl_2_, 400 MHz). Figure S21: ^13^C NMR spectrum of **AQM2** (CD_2_Cl_2_, 75 MHz). Figure S22: ^1^H NMR spectrum of **AQM3** (CD_2_Cl_2_, 300 MHz). Figure S23: ^13^C NMR spectrum of **AQM3** (CD_2_Cl_2_, 75 MHz).
