# Supplementary material for: New Quinoid Bio-Inspired Materials Using Para-Azaquinodimethane Moiety
Source: Molecules. 2023 Dec 28;29(1):186. doi: 10.3390/molecules29010186 (PMC10780065; doi:10.3390/molecules29010186)
Supplement: Supplementary file 1 [file molecules-29-00186-s001.zip › molecules-2748868-supplementary.pdf]

# Supporting Information


1. **Optical properties……………………………………………………2**
    *1a) Comparison of UV spectra in different solvents………………………………………...2*
    *1b) Absorption spectra of non-quinoidal vs quinoidal intermediates…………………….3*
    *1c) Calculation of molar extinction coefficients……………………………………………….3*

2. **Thermal properties……………………………………………..…5**

3. **Theoretical calculations……………………………………...…6**

4. **NMR spectra…………………………………………………….....14**

5. **References………………………………………………………20**

## 1. Optical properties

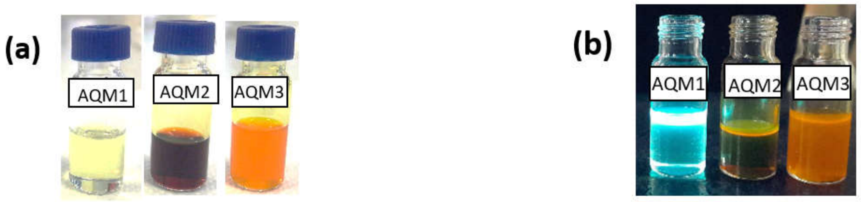


**Figure S1:** Photos showing the colors of p-AQM molecules in chloroform solutions (a) and under UV light of 254 nm (b).

*1a) Comparison of UV spectra in different solvents*

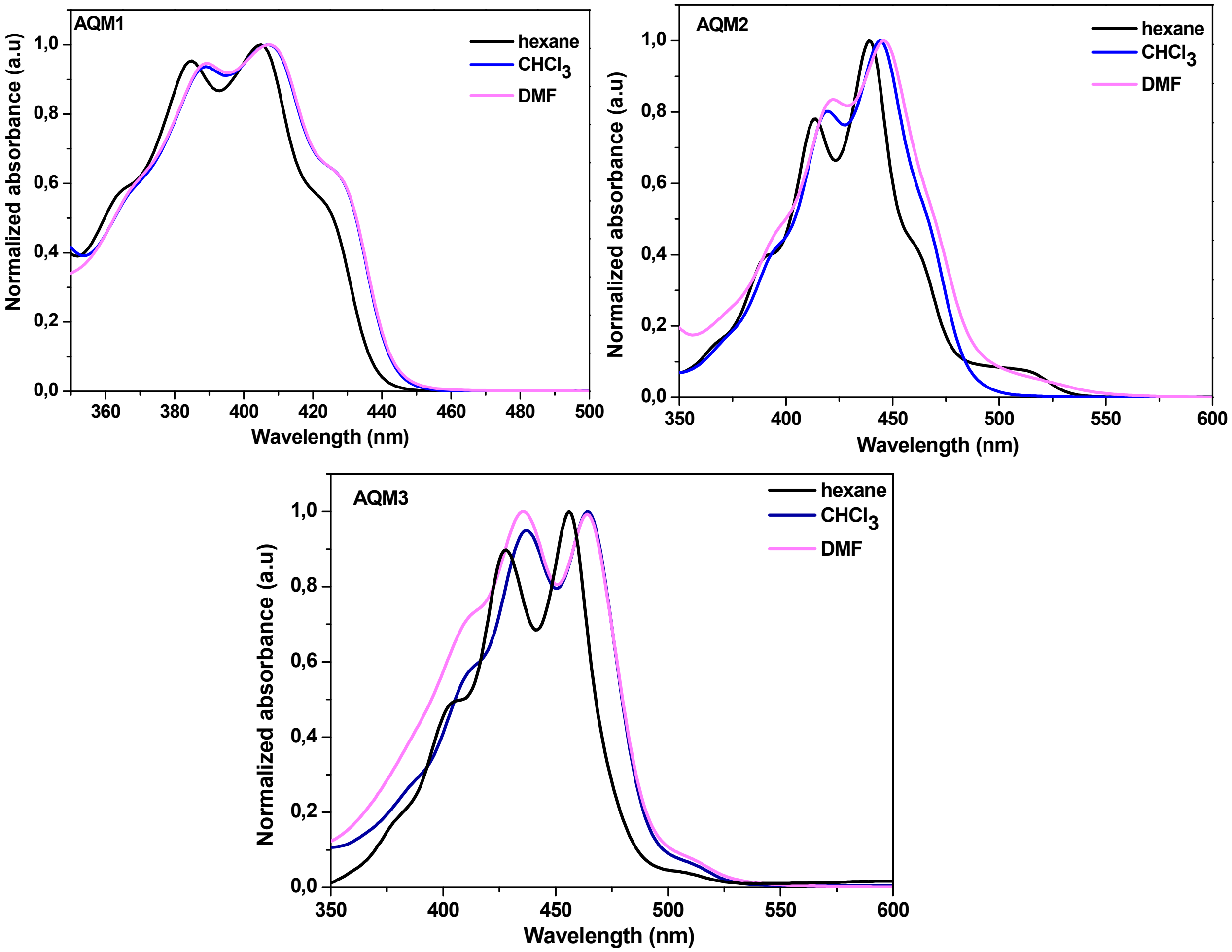


**Figure S2**: Normalized UV/visible absorption spectra of **AQM1**, **AQM2** and **AQM3** in different solvents

**Table S1**: Maximum absorption wavelengths of **AQM 1-3** in different solvents

| | $\lambda_{max}$[nm] (AQM 1) | $\lambda_{max}$ [nm] (AQM 2) | $\lambda_{max}$ [nm] (AQM 3) |
|---|---|---|---|
| **Hexane** | 405 | 440 | 456 |
| **$CHCl_3$** | 407 | 444 | 465 |
| **DMF** | 407 | 446 | 465 |

*1b) Absorption spectra of non-quinoidal vs quinoidal molecules*

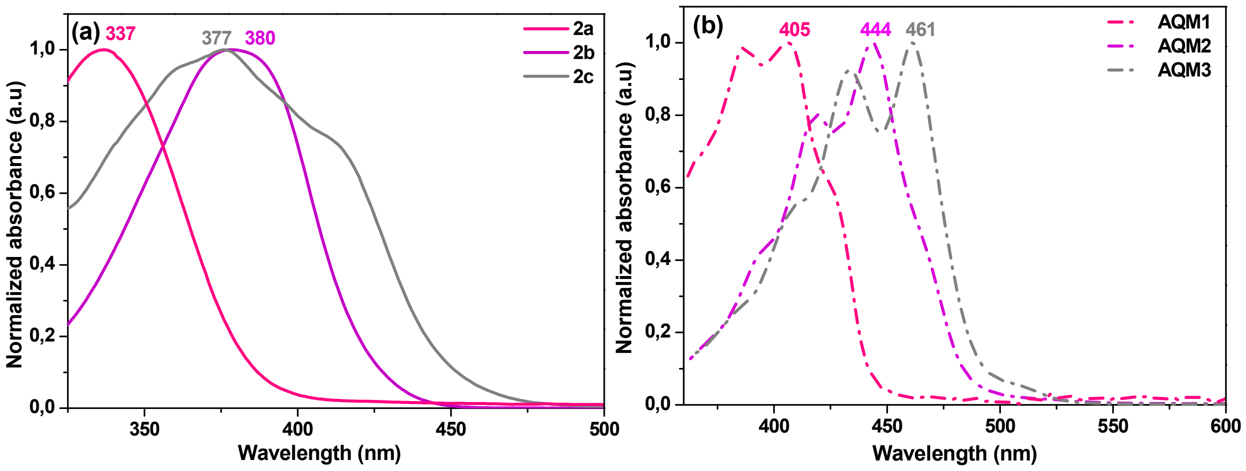


**Figure S3:** Comparison of normalized UV/visible absorption spectra of (a) non-quinoidal intermediates **2a-c,** and (b) p-AQM molecules in $10^{-5}$ M THF solution .

*1c) Calculation of molar extinction coefficients*

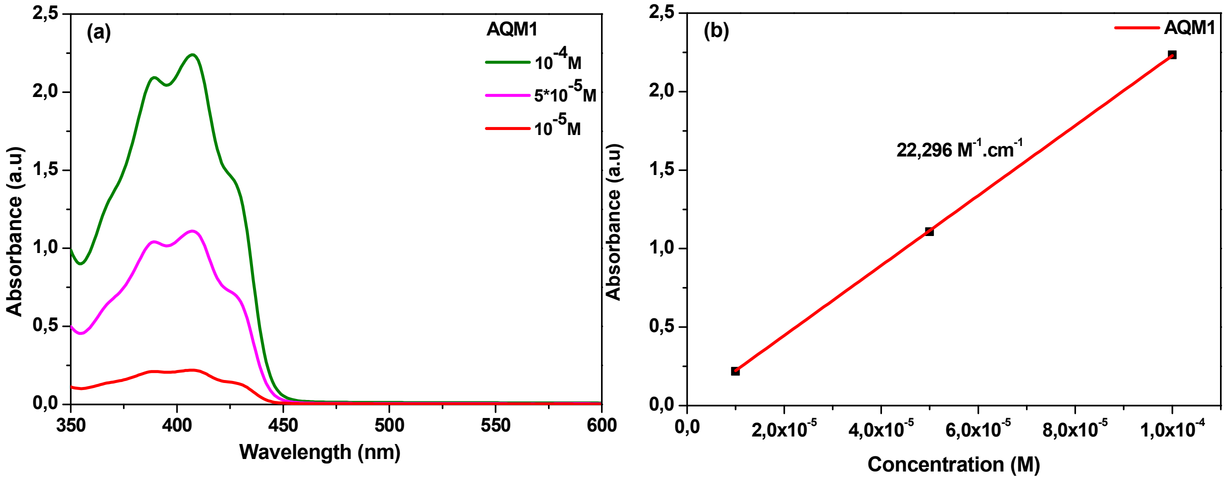


**Figure S4**: (a) UV/visible absorption spectra of **AQM1** at different concentrations in $CHCl_3$
(b) Absorbance vs Concentration plot of **AQM1** in $CHCl_3$

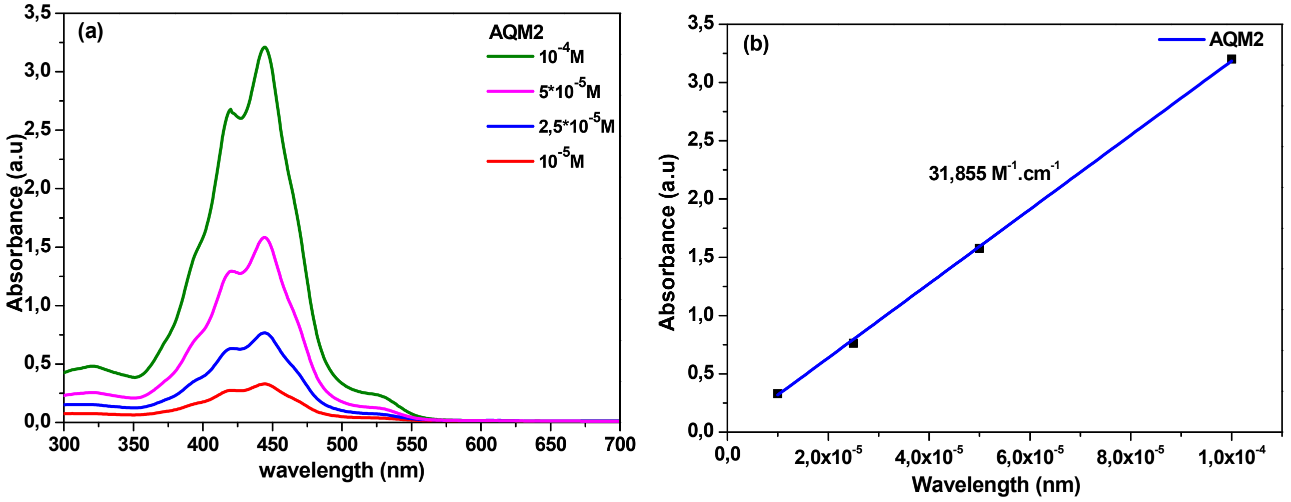


**Figure S5**: (a) UV/visible absorption spectra of **AQM2** at different concentrations in $CHCl_3$
(b) Absorbance vs Concentration plot of **AQM2** in $CHCl_3$

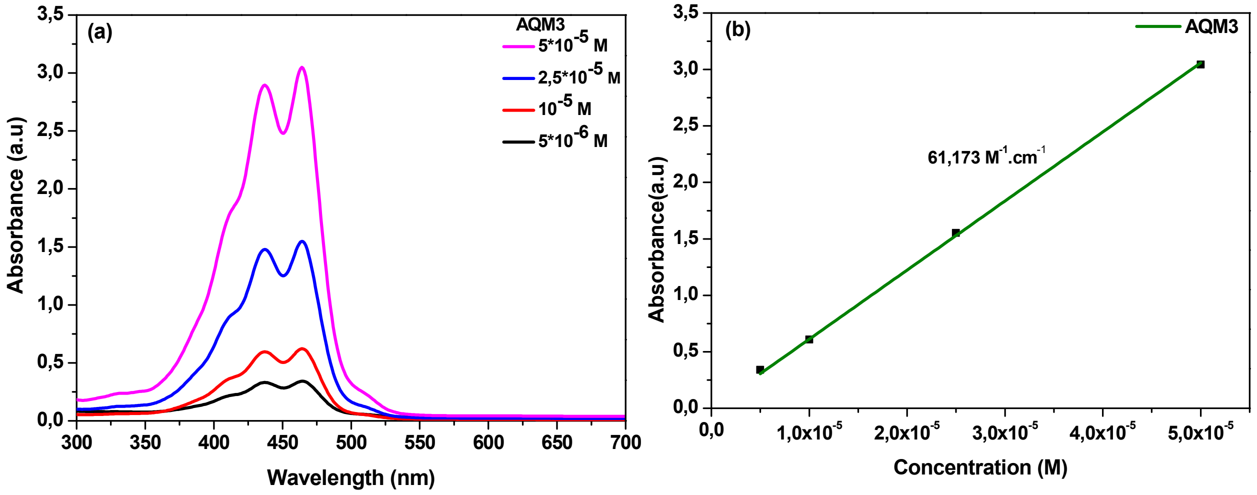


**Figure S6**: (a) UV/visible absorption spectra of **AQM3** at different concentrations in $CHCl_3$
(b) Absorbance vs Concentration plot of **AQM3** in $CHCl_3$

## 2. Thermal properties

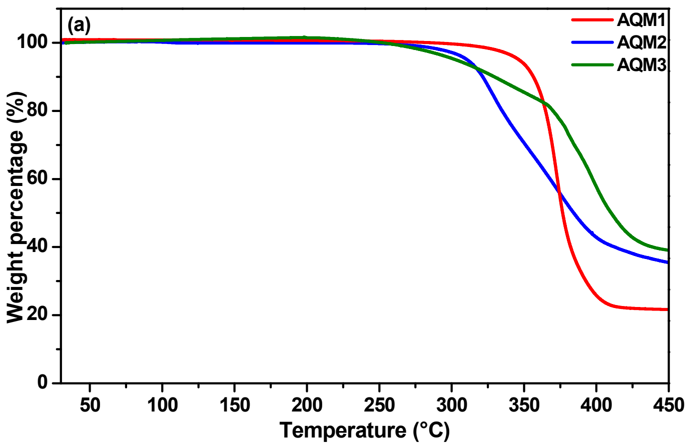


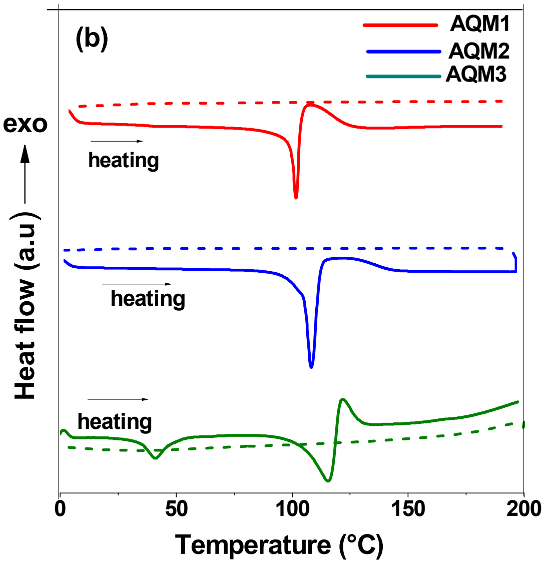


**Figure S7**: (a): TGA curves under nitrogen at 10 °C /min scan rate. (b): DSC curves under nitrogen: 1st heating (full line) and cooling cycles (dashed line) at 10 °C/min scan rate

**Table S2**: Thermal properties of p-AQM molecules

| | Td (°C) | Tm[a] (°C) | Tm[b] (°C) |
|---|---|---|---|
| **AQM1** | 340 | 105 | 109 |
| **AQM2** | 312 | 110 | 103 |
| **AQM3** | 292 | 40, 115 | 117 |

[a]: Measured by DSC
[b]: Measured by capillary method

## 3. Theoretical calculations

**AQM1** and **AQM2** can exist as Z and E isomers depending on the configuration of the double bond connecting the azaquinodimethane unit with the substituents (see for example **Figure S8** ). Only the symmetric isomers with the same E or Z conformations on the left and right sides of the molecule are considered for simplicity.

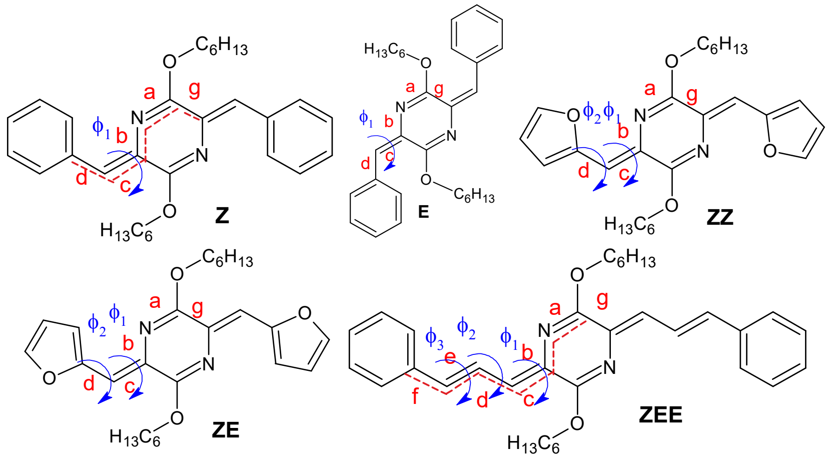


**Figure S8:** E, Z, ZZ, ZE and ZEE isomers displayed for **AQM1-3**

For **AQM3** due to the presence of several double bond between the phenyl groups and the central unit we calculated 8 isomers and concluded that the isomer labeled as ZEE is the most stable. Furthermore and considering the energies difference with the other isomer, i. e. more than 10 kJ/mol, the ZEE isomer is the only one that we consider to calculate the absorption spectra of this molecule. All the PBE0, B3LYP and ωB97X-D XCF give the same energetic ordering of the different isomer: Z < E for **AQM1**, ZE < ZZ < EE < EZ for **AQM2** and ZEE < EEE **<** ZEZ < ZZE < EEZ < ZZZ < EZE < EZZ for **AQM3** (See **Table S3**)

**Table S3:** Relatives energies (in $kJ.mol^{-1}$) of the isomers of AQM1, AQM2 and AQM3 compounds calculated at the PBE0/6-311G(d,p) and B3LYP/6-311G(d,p) levels.

| **AQM1** | **Z** | **E** | | | | | | |
|---|---|---|---|---|---|---|---|---|
| PBE0 | 0 | 54.9 | | | | | | |
| B3LYP | 0 | 54.1 | | | | | | |
| ωB97XD | 0 | 40.8 | | | | | | |
| **AQM2** | **ZE** | **EE** | **ZZ** | **EZ** | | | | |
| PBE0 | 0 | 50.1 | 14.9 | 60.3 | | | | |
| B3LYP | 0 | 49.3 | 13.6 | 58.9 | | | | |
| ωB97XD | 0 | 50.0 | 21.1 | 57.8 | | | | |
| **AQM3** | **EEE** | **EEZ** | **ZEE** | **ZEZ** | **EZE** | **ZZE** | **ZZZ** | **EZZ** |
| PBE0 | 23.8 | 56.9 | 0 | 29.8 | 86.5 | 30.1 | 79.3 | 117.9 |
| B3LYP | 24.0 | 58.3 | 0 | 31.9 | 88.1 | 33.6 | 91.7 | 128.5 |
| ωB97XD | 21.3 | 47.0 | 0 | 19.1 | 50.9 | 18.2 | 27.3 | 48.9 |

**AQM1 (Z) AQM1 (E) AQM2 (ZE) AQM2 (EE) AQM3 (ZEE)**

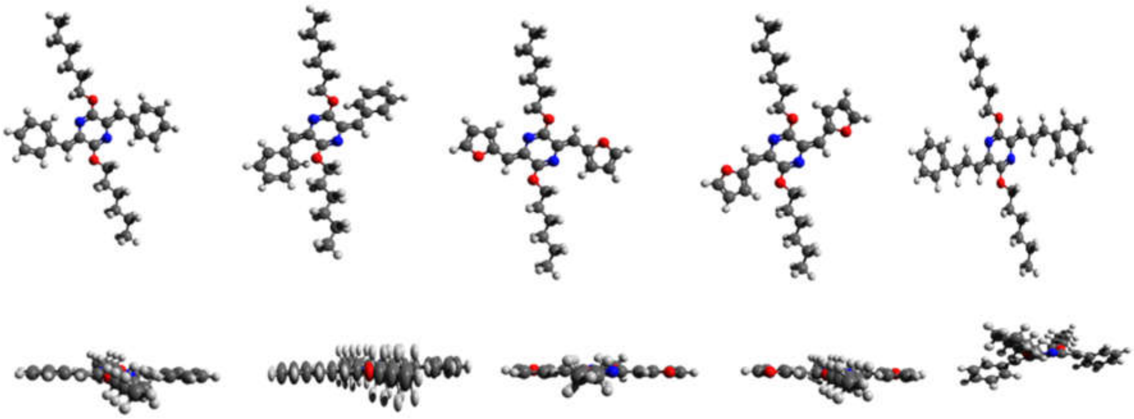

**Figure S9.** Optimized geometries of the isomers of AQM1, AQM2 and AQM3 (ZEE) compounds (PBE0/6-311G(d,p) calculations) (top view and side view).

**Table S4:** Geometric parameters of the isomers of AQM1, AQM2 and AQM3 compounds calculated at the PBE0/6-311G(d,p) and B3LYP/6-311G(d,p) levels.

| | a | B | c | D | e | f | g | ϕ1 | ϕ2 | ϕ3 | BLA |
|---|---|---|---|---|---|---|---|---|---|---|---|
| | | | | | | **AQM1** | | | | | |
| **PBE0** | | | | | | | | | | | |
| Z | 1.285 | 1.382 | 1.361 | 1.450 | | | 1.466 | 0.0 | | | 0.110 |
| E | 1.282 | 1.391 | 1.357 | 1.458 | | | 1.469 | 173.4 | | | 0.120 |
| **B3LYP** | | | | | | | | | | | |
| Z | 1.288 | 1.389 | 1.368 | 1.455 | | | 1.473 | 0.0 | | | 0.111 |
| E | 1.285 | 1.398 | 1.361 | 1.462 | | | 1.476 | 173.2 | | | 0.122 |
| **ωB97XD** | | | | | | | | | | | |
| Z | 1.280 | 1.391 | 1.351 | 1.461 | | | 1.476 | 0.0 | | | 1.127 |
| E | 1.278 | 1.401 | 1.345 | 1.472 | | | 1.482 | 174.3 | | | 0.140 |
| | | | | | | **AQM2** | | | | | |
| **PBE0** | | | | | | | | | | | |
| ZE | 1.287 | 1.381 | 1.363 | 1.423 | | | 1.460 | 0 | 180.0 | | 0.096 |
| EE | 1.284 | 1.386 | 1.367 | 1.458 | | | 1.458 | 180.0 | 180.0 | | 0.108 |
| ZZ | 1.285 | 1.379 | 1.363 | 1.423 | | | 1.463 | 0.4 | 0.3 | | 0.098 |
| EZ | 1.284 | 1.387 | 1.363 | 1.426 | | | 1.465 | 172.1 | 11.6 | | 0.102 |
| **B3LYP** | | | | | | | | | | | |
| ZE | 1.290 | 1.387 | 1.367 | 1.426 | | | 1.466 | 0.0 | 180.0 | | 0.098 |
| EE | 1.287 | 1.393 | 1.372 | 1.428 | | | 1.463 | 178.9 | 179.0 | | 0.098 |
| ZZ | 1.288 | 1.385 | 1.367 | 1.426 | | | 1.468 | 0.0 | 0.0 | | 0.099 |
| EZ | 1.288 | 1.394 | 1.367 | 1.429 | | | 1.470 | 171.8 | 11.4 | | 0.103 |
| **ωB97XD** | | | | | | | | | | | |
| ZE | 1.281 | 1.389 | 1.352 | 1.435 | | | 1.470 | 0.0 | 180.0 | | 0.115 |
| EE | 1.278 | 1.397 | 1.355 | 1.439 | | | 1.469 | 177.6 | 174.8 | | 0.118 |
| ZZ | 1.279 | 1.388 | 1.352 | 1.435 | | | 1.473 | 0.0 | 0.0 | | 0.116 |
| EZ | 1.279 | 1.398 | 1.348 | 1.444 | | | 1.479 | 172.1 | 26.3 | | 0.127 |

| AQM3 | | | | | | | | | | |
|---|---|---|---|---|---|---|---|---|---|---|
| **PBE0** | | | | | | | | | | | |
| EEE | 1.285 | 1.383 | 1.370 | 1.425 | 1.355 | 1.453 | 1.461 | 180.0 | 180.0 | 180.0 | 0.094 |
| EEZ | 1.284 | 1.384 | 1.369 | 1.430 | 1.357 | 1.460 | 1.463 | 175.9 | 174.0 | 7.0 | 0.097 |
| ZEE | 1.286 | 1.380 | 1.366 | 1.426 | 1.353 | 1.454 | 1.461 | 0.0 | 180.0 | 180.0 | 0.095 |
| ZEZ | 1.286 | 1.388 | 1.366 | 1.430 | 1.355 | 1.461 | 1.463 | 2.8 | 175.7 | 6.2 | 0.100 |
| EZE | 1.284 | 1.386 | 1.368 | 1.437 | 1.350 | 1.456 | 1.464 | 173.5 | 26.0 | 177.0 | 0.102 |
| ZZE | 1.288 | 1.378 | 1.369 | 1.434 | 1.355 | 1.455 | 1.461 | 0.1 | 0.9 | 179.9 | 0.095 |
| ZZZ | 1.285 | 1.378 | 1.362 | 1.444 | 1.352 | 1.459 | 1.467 | 1.4 | 40.7 | 8.8 | 0.104 |
| EZZ | 1.283 | 1.388 | 1.359 | 1.449 | 1.347 | 1.463 | 1.468 | 176.6 | 46.2 | 2.4 | 0.112 |
| **B3LYP** | | | | | | | | | | | |
| EEE | 1.288 | 1.390 | 1.374 | 1.428 | 1.359 | 1.457 | 1.466 | 180.0 | 180.0 | 180.0 | 0.095 |
| EEZ | 1.288 | 1.391 | 1.373 | 1.433 | 1.360 | 1.465 | 1.468 | 175.9 | 175.3 | 6.8 | 0.099 |
| ZEE | 1.290 | 1.387 | 1.371 | 1.429 | 1.356 | 1.458 | 1.466 | 0.0 | 180.0 | 180.0 | 0.096 |
| ZEZ | 1.289 | 1.387 | 1.370 | 1.433 | 1.358 | 1.465 | 1.468 | 2.8 | 176.3 | 6.0 | 0.099 |
| EZE | 1.287 | 1.393 | 1.373 | 1.441 | 1.354 | 1.460 | 1.469 | 172.8 | 23.6 | 176.4 | 0.103 |
| ZZE | 1.292 | 1.385 | 1.373 | 1.437 | 1.358 | 1.460 | 1.467 | 0.2 | 1.0 | 179.9 | 0.096 |
| ZZZ | 1.288 | 1.385 | 1.364 | 1.449 | 1.354 | 1.464 | 1.473 | 1.0 | 42.7 | 8.6 | 0.107 |
| EZZ | 1.286 | 1.396 | 1.362 | 1.454 | 1.350 | 1.467 | 1.473 | 178.1 | 49.2 | 2.7 | 0.115 |
| **ωB97XD** | | | | | | | | | | | |
| EEE | 1.279 | 1.396 | 1.357 | 1.441 | 1.345 | 1.465 | 1.473 | 180.0 | 180.0 | 180.0 | 0.117 |
| EEZ | 1.278 | 1.396 | 1.355 | 1.446 | 1.346 | 1.472 | 1.473 | 176.7 | 170.8 | 5.7 | 0.120 |
| ZEE | 1.280 | 1.392 | 1.353 | 1.440 | 1.344 | 1.466 | 1.472 | 0.0 | 180.0 | 180.0 | 0.117 |
| ZEZ | 1.280 | 1.392 | 1.352 | 1.445 | 1.345 | 1.473 | 1.473 | 2.0 | 173.4 | 4.9 | 0.120 |
| EZE | 1.278 | 1.401 | 1.349 | 1.457 | 1.340 | 1.468 | 1.483 | 171.9 | 38.2 | 176.5 | 0.130 |
| ZZE | 1.282 | 1.387 | 1.356 | 1.449 | 1.347 | 1.466 | 1.470 | 0.0 | 0.4 | 180.0 | 0.115 |
| ZZZ | 1.280 | 1.386 | 1.350 | 1.456 | 1.344 | 1.469 | 1.476 | 1.8 | 42.2 | 5.6 | 0.122 |
| EZZ | 1.278 | 1.400 | 1.346 | 1.463 | 1.341 | 1.473 | 1.478 | 175.2 | 44.8 | 0.8 | 0.132 |

*Energies of the HOMO and LUMO orbitals*

**Table S5:** Energies (in eV) of HOMO, LUMO and HOMO/LUMO gap of the isomers of AQM1, AQM2 and AQM3 compounds calculated at the PBE0/6-311G(d,p), B3LYP/6-311G(d,p) and ωB97XD/6-311G(d,p) levels.

| | HOMO | LUMO | HOMO/LUMO gap |
|---|---|---|---|
| | **AQM1** | | |
| PBE0 | | | |
| Z | -5.646 | -2.382 | 3.263 |
| E | -5.738 | -2.261 | 3.477 |
| B3LYP | | | |
| Z | -5.435 | -2.474 | 2.961 |
| E | -5.516 | -2.474 | 3.04 |
| ωB97XD | | | |
| Z | -7.230 | -0.827 | 6.403 |
| E | -7.541 | -0.443 | 7.098 |
| | **AQM2** | | |
| PBE0 | | | |
| ZE | -5.319 | -2.344 | 2.975 |

| | | | |
|---|---|---|---|
| EE | -5.239 | -2.344 | 2.895 |
| ZZ | -5.277 | -2.283 | 2.994 |
| EZ | -5.278 | -2.260 | 3.018 |
| B3LYP | | | |
| ZE | -5.141 | -2.460 | 2.681 |
| EE | -5.066 | -2.462 | 2.603 |
| ZZ | -5.099 | -2.400 | 2.698 |
| EZ | -5.099 | -2.384 | 2.715 |
| ωB97XD | | | |
| ZE | -6.940 | -0.823 | 6.116 |
| EE | -6.855 | -0.819 | 6.036 |
| ZZ | -6.879 | -0.763 | 6.116 |
| EZ | -7.085 | -0.608 | 6.477 |
| | **AQM3** | | |
| PBE0 | | | |
| EEE | -5.241 | -2.632 | 2.609 |
| EEZ | -5.370 | -2.575 | 2.795 |
| ZEE | -5.305 | -2.574 | 2.734 |
| ZEZ | -5.592 | -2.540 | 3.051 |
| EZE | -5.358 | -2.609 | 2.748 |
| ZZE | -5.321 | -2.679 | 2.642 |
| ZZZ | -5.592 | -2.437 | 3.155 |
| EZZ | -5.585 | -2.381 | 3.203 |
| B3LYP | | | |
| EEE | -5.053 | -2.714 | 2.340 |
| EEZ | -5.177 | -2.680 | 2.497 |
| ZEE | -5.114 | -2.658 | 2.456 |
| ZEZ | -5.239 | -2.647 | 2.592 |
| EZE | -5.150 | -2.713 | 2.437 |
| ZZE | -5.128 | -2.760 | 2.367 |
| ZZZ | -5.391 | -2.519 | 7.910 |
| EZZ | -5.390 | -2.494 | 2.896 |
| ωB97XD | | | |
| EEE | -6.783 | -1.095 | 5.691 |
| EEZ | -6.969 | -0.959 | 6.010 |
| ZEE | -6.871 | -1.020 | 5.851 |
| ZEZ | -7.046 | -0.906 | 6.140 |
| EZE | -7.273 | -0.705 | 6.568 |
| ZZE | -6.903 | -1.133 | 5.770 |
| ZZZ | -7.265 | -0.781 | 6.484 |
| EZZ | -7.376 | -0.617 | 6.759 |

**AQM1**

**Z** HOMO | LUMO — **E** HOMO | LUMO

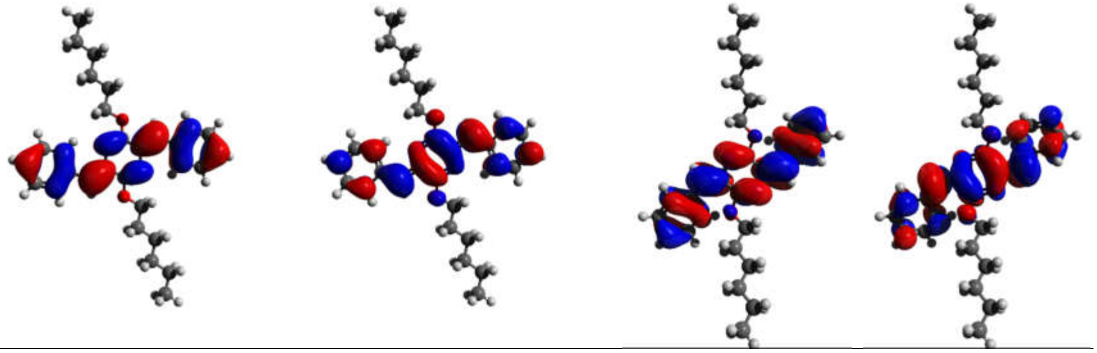

**AQM2**

**ZE** HOMO | LUMO — **EE** HOMO | LUMO

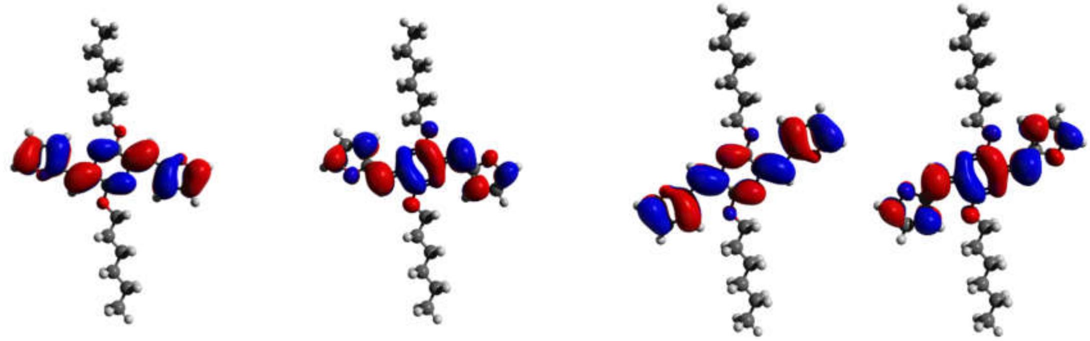

**AQM3 (ZEE isomer)**

HOMO | LUMO

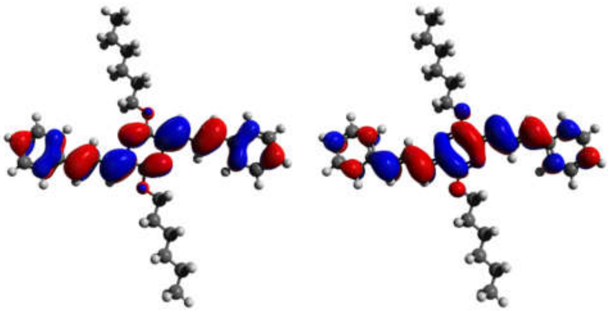

**Figure S10.** HOMO and LUMO orbitals for AQM1, AQM2 and AQM3 obtained at the PBE0/6-311G(d,p) level of calculations

To get a first qualitative idea of the TDDFT performance with respect to this family of compounds, we startby computing their TDDFT vertical absorption spectra in chloroform solvent from their ground-state optimized structures (**Figure S11**) either with PBE0, B3LYP or ωB97XD functionals. The results are compiled in **Table S6**. The Z, ZE and ZEE isomers of **AQM1**, **AQM2** and **AQM3** were used for this study.

It is clear that CAM-B3LYP, M062X and ωB97XD functionals provide almost similar results and the better agreement with experimental data for the lowest maximum absorption. The PBE0, B3LYP and M06 are dismissed due to the large red-shift in the absorption energies. Following this study, we selected

the ωB97XD functional to calculate the excited state energies in combination with the geometries calculated with PBE0. The calculated $S_0 \rightarrow S_1$ energies using the combination are found to be 465 nm for AQM3 (463 nm exptl), 408 nm for AQM1 (400 nm exptl) and 434 nm for AQM2 (446 nm exptl)

**Table S6:** Benchmark on transitions for **AQM3** (ZEE isomer), **AQM1** (Z isomer) and **AQM2 (ZE isomer)** compounds. The ground state geometries were computed using the 6-311G(d,p) basis-set using PBE0, B3LYP and ωB97XD functionals.

| | AQM3 | | AQM1 | | AQM2 | |
|---|---|---|---|---|---|---|
| | PBE0/6-311G(d,p) | | | | | |
| | $S_0 \rightarrow S_1$ | *f* | $S_0 \rightarrow S_1$ | *f* | $S_0 \rightarrow S_1$ | *f* |
| PBE0 | 510 | 2.494 | 427 | 1.681 | 460 | 1.513 |
| B3LYP | 523 | 2.443 | 437 | 1.645 | 469 | 1.481 |
| CAM-B3LYP | 470 | 2.583 | 402 | 1.751 | 435 | 1.561 |
| M06 | 518 | 2.448 | 435 | 1.644 | 467 | 1.483 |
| M062X | 468 | 2.561 | 399 | 1.746 | 433 | 1.554 |
| ωB97XD | 465 | 2.588 | 400 | 1.751 | 434 | 1.564 |
| Exptl | 463 | | 408 | | 446 | |
| | B3LYP/6-311G(d,p) | | | | | |
| | $S_0 \rightarrow S_1$ | *f* | $S_0 \rightarrow S_1$ | *f* | $S_0 \rightarrow S_1$ | *f* |
| PBE0 | 512 | 2.502 | 429 | 1.687 | 463 | 1.527 |
| B3LYP | 526 | 2.451 | 439 | 1.651 | 472 | 1.495 |
| CAM-B3LYP | 472 | 2.592 | 403 | 1.759 | 437 | 1.575 |
| M06 | 521 | 2.455 | 437 | 1.651 | 470 | 1.496 |
| M062X | 470 | 2.570 | 400 | 1.754 | 435 | 1.568 |
| ωB97XD | 468 | 2.597 | 401 | 1.759 | 436 | 1.589 |
| Exptl | 463 | | 408 | | 446 | |
| | ωB97XD /6-311G(d,p) | | | | | |
| | $S_0 \rightarrow S_1$ | *f* | $S_0 \rightarrow S_1$ | *f* | $S_0 \rightarrow S_1$ | *f* |
| PBE0 | 480 | 2.433 | 412 | 1.653 | 443 | 1.469 |
| B3LYP | 493 | 2.376 | 422 | 1.615 | 452 | 1.436 |
| CAM-B3LYP | 438 | 2.555 | 386 | 1.734 | 415 | 1.527 |
| M06 | 487 | 2.394 | 420 | 1.619 | 449 | 1.442 |
| M062X | 437 | 2.528 | 383 | 1.728 | 414 | 1.518 |
| ωB97XD | 434 | 2.564 | 394 | 1.736 | 414 | 1.532 |
| Exptl | 463 | | 408 | | 446 | |

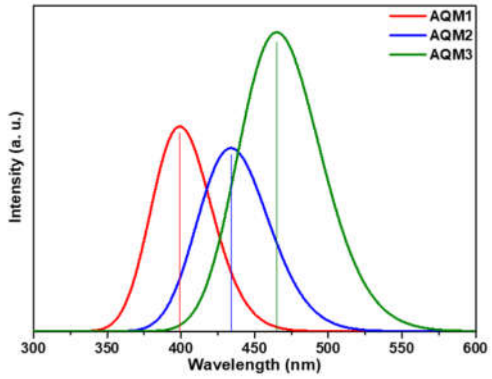


**Figure S11**: TD-DFT calculations of the absorption spectra of **AQM1** (Z), **AQM2** (ZE) and **AQM3** (ZEE)

**Table S7:** Lowest electronic transition energies calculated for AQM3, AQM1 and AQM2 compounds at the ωB97XD/6-311G(d,p) PBE1PBE/6-311G(d,p).

| | $S_0 \rightarrow S_1$ | *f* |
|---|---|---|
| | **AQM3** | |
| EEE | 482 | 2.569 |
| EEZ | 456 | 2.718 |
| ZEE | 465 | 2.588 |
| EZE | 475 | 2.610 |
| ZEZ | 443 | 2.798 |
| ZZZ | 427 | 2.903 |
| EZZ | 428 | 2.898 |
| ZZE | 492 | 2.521 |
| Exptl | 463 | |
| | **AQM1** | |
| Z | 400 | 1.7512 |
| E | 383 | 1.555 |
| Exptl | 408 | |
| | **AQM2** | |
| ZE | 434 | 1.564 |
| EE | 442 | 1.797 |
| ZZ | 427 | 1.711 |
| EZ | 431 | 1.534 |
| Exptl | 446 | |

**Table S8:** Lowest electronic transition energies in absorption and emission calculated for **AQM3** (ZEE isomer), **AQM1** (Z isomer) and **AQM2** (ZE isomer) compounds at the ωB97XD/6-311G(d,p) PBE1PBE/6-311G(d,p) level.

| | **Absorption** | | **Emission** | |
|---|---|---|---|---|
| | $S_0 \rightarrow S_1$ | *f* | $S_1 \rightarrow S_0$ | *f* |
| **AQM1** | 400 | 1.751 | 481 | 2.250 |
| **AQM2** | 434 | 1.564 | 512 | 2.420 |
| **AQM3** | 465 | 2.588 | 626 | 1.981 |

*Redox potentials*

DFT calculations were also performed to determine the redox potentials in chloroform according to the thermodynamic cycle shown below:

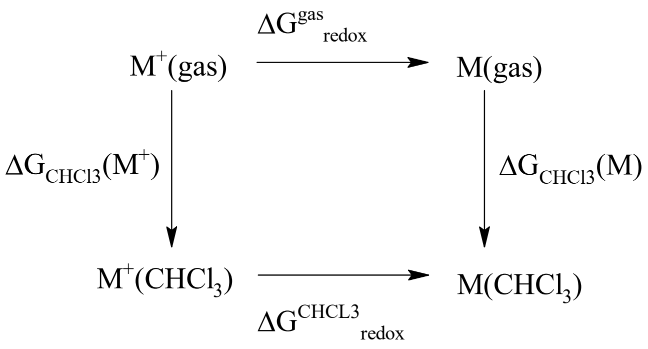


$$\Delta G^{CHCL3}_{redox} = \Delta G^{gas}_{redox} + \Delta G^{CHCL3}(M^+) - \Delta G^{CHCL3}(M)$$

$$\Delta G^{CHCL3}_{redox} = \Delta G^{gas}_{redox} + \Delta\Delta G_{sol}$$

Where

$$\Delta G^{gas}_{redox} = G^{gas}(M) - G^{gas}(M^+)$$

And

$$\Delta\Delta G_{sol} = \Delta G^{CHCL3}(M^+) - \Delta G^{CHCL3}(M)$$

And the redox potential is related to $\Delta G_{redox}(CHCl3)$ as $\Delta G^{CHCL3}_{redox} = -FE^{\circ}_{redox}$

Where *F* is the Faraday constant, 23.06 kcal.mol$^{-1}$ V$^{-1}$. The standard hydrogen electrode potential, $V_{SHE}$ was taken as 4.43 eV [1].

From the lowest isomers of **AQM1**, **AQM2** and **AQM3**, i.e. Z, ZE and ZEE isomers respectively, the redox potentials were calculated in chloroform according to the thermodynamic cycle shown above. Results are gathered in **Table S9** and compared with the experimental ones.

**Table S9.** Computed redox potentials vs. experimental ones of the **AQM1**, **AQM2** and **AQM3** compounds. For each compound, calculation were performed on the most stable isomer at the PBE0/6-311(g,d) level.

| | **AQM1** | **AQM2** | **AQM3** |
|---|---|---|---|
| Calc E (V vs SCE) | 1.00 | 0.72 | 0.67 |
| Exp E (V vs SCE) | 0.72 | 0.64 | 0.72 |

The calculated results obtained for **AQM2** and **AQM3** are in relatively good agreement with the experimental ones measured by cyclic voltammetry with an error less than 0.08 V. Concerning **AQM1** the agreement is less satisfactory and the calculated error is 0.28 V.

## 4. NMR spectra

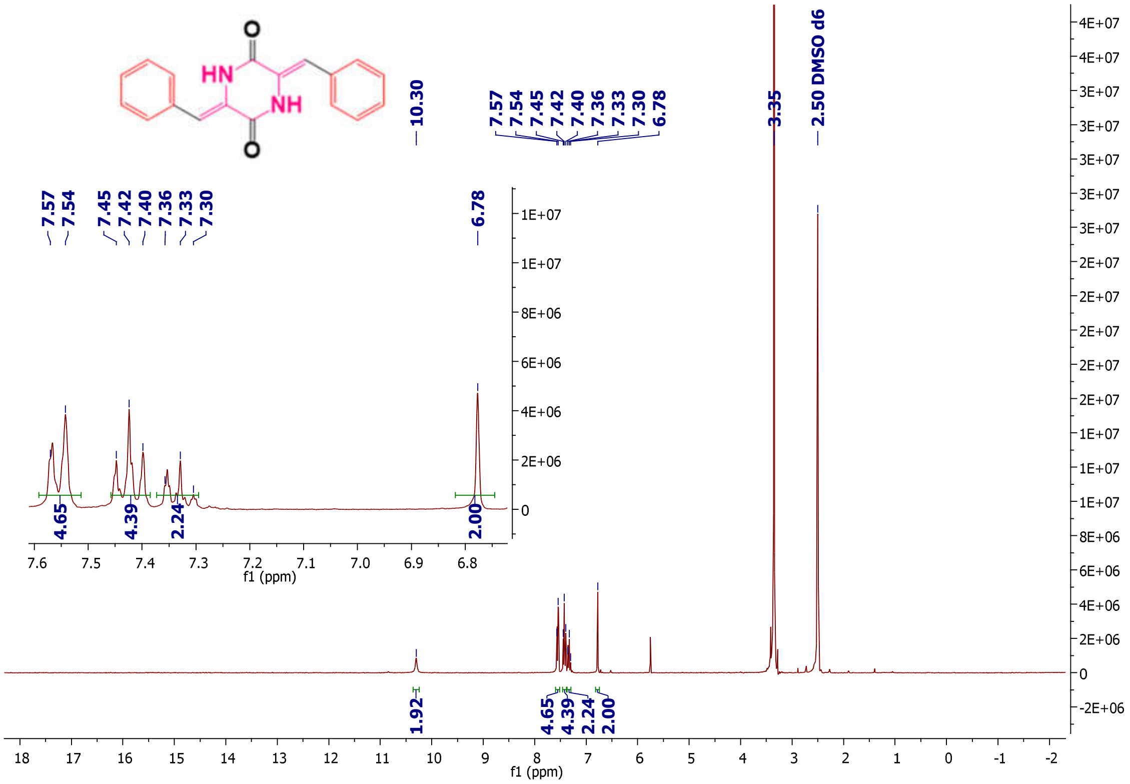


**Figure S12**: [1] H NMR spectrum of **2a** (DMSO $d_6$, 300 MHz)

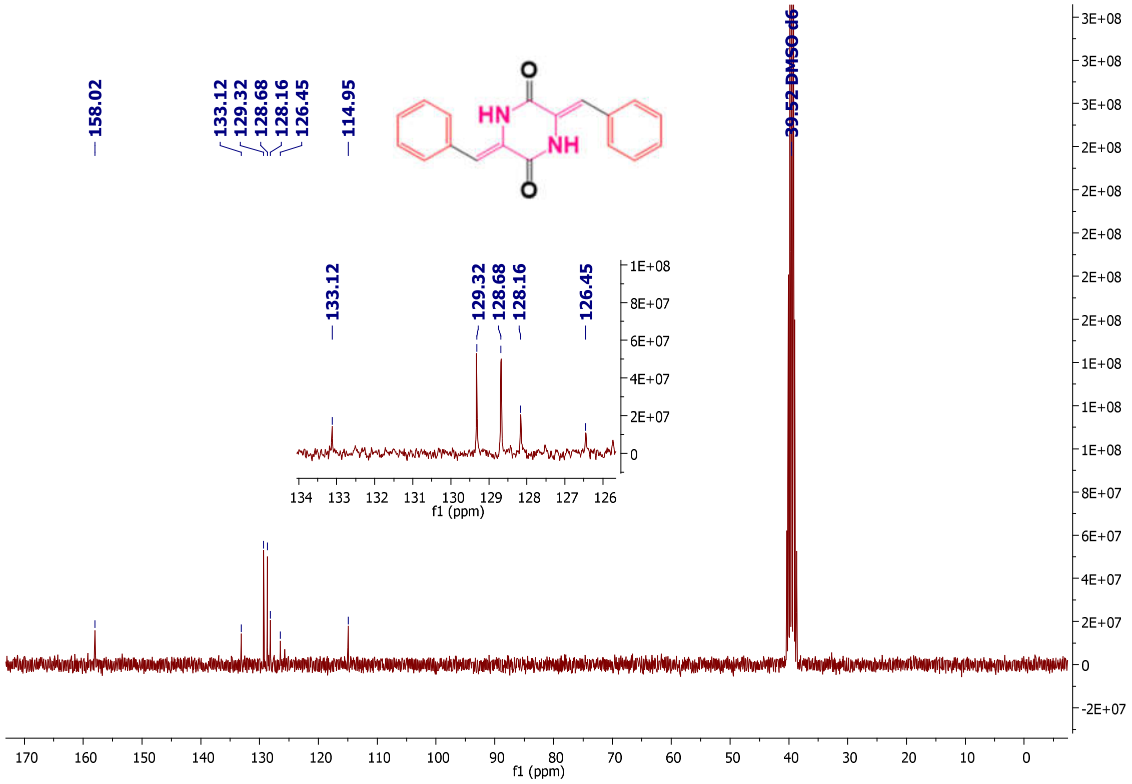


**Figure S13**: $^{13}$C NMR spectrum of **2a** (DMSO $d_6$, 75 MHz)

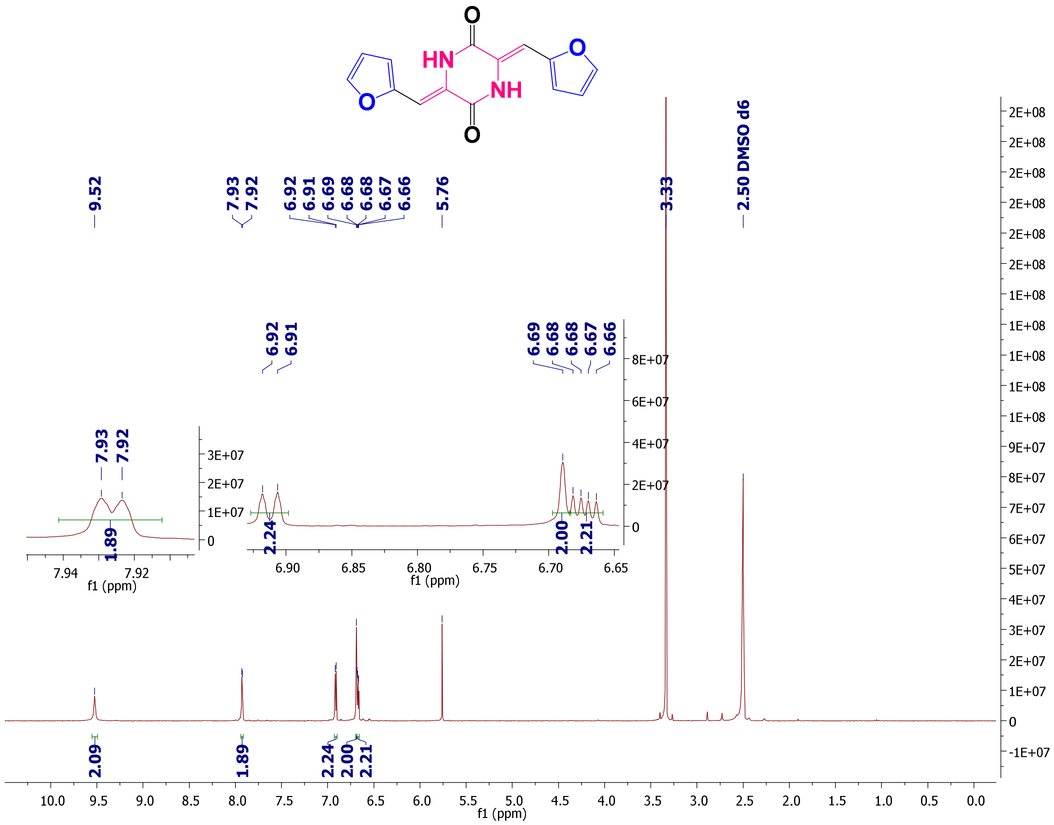

**Figure S14**: $^{1}$H NMR spectrum of **2b** (DMSO $d_6$, 300 MHz)

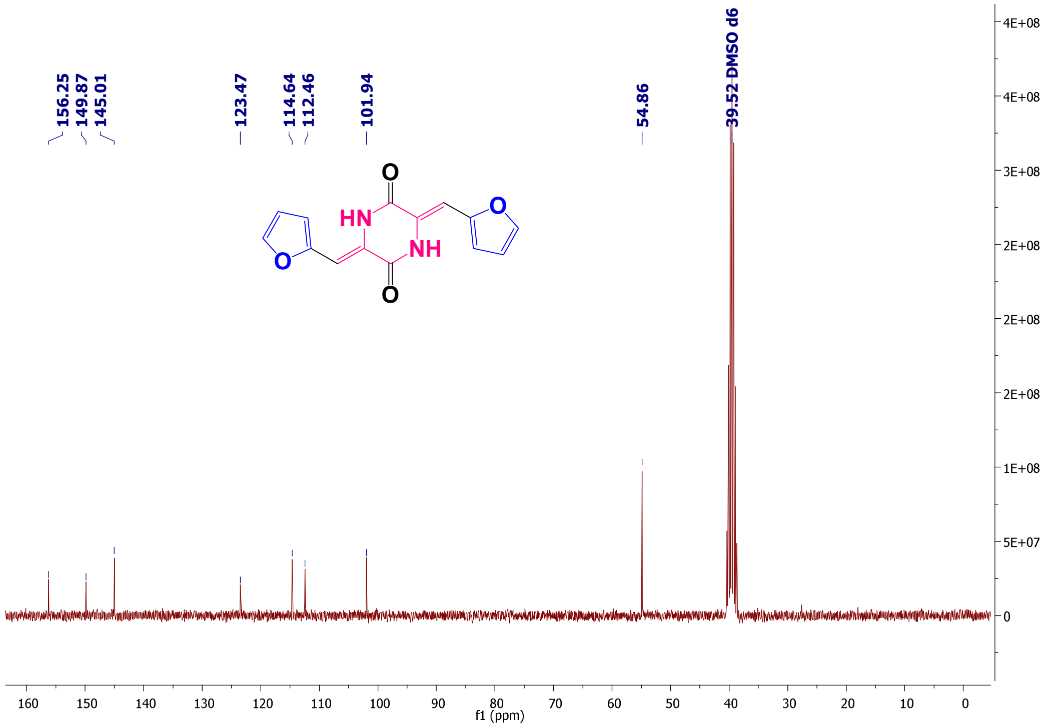

**Figure S15**: $^{13}$C NMR spectrum of **2b** (DMSO $d_6$, 75 MHz)

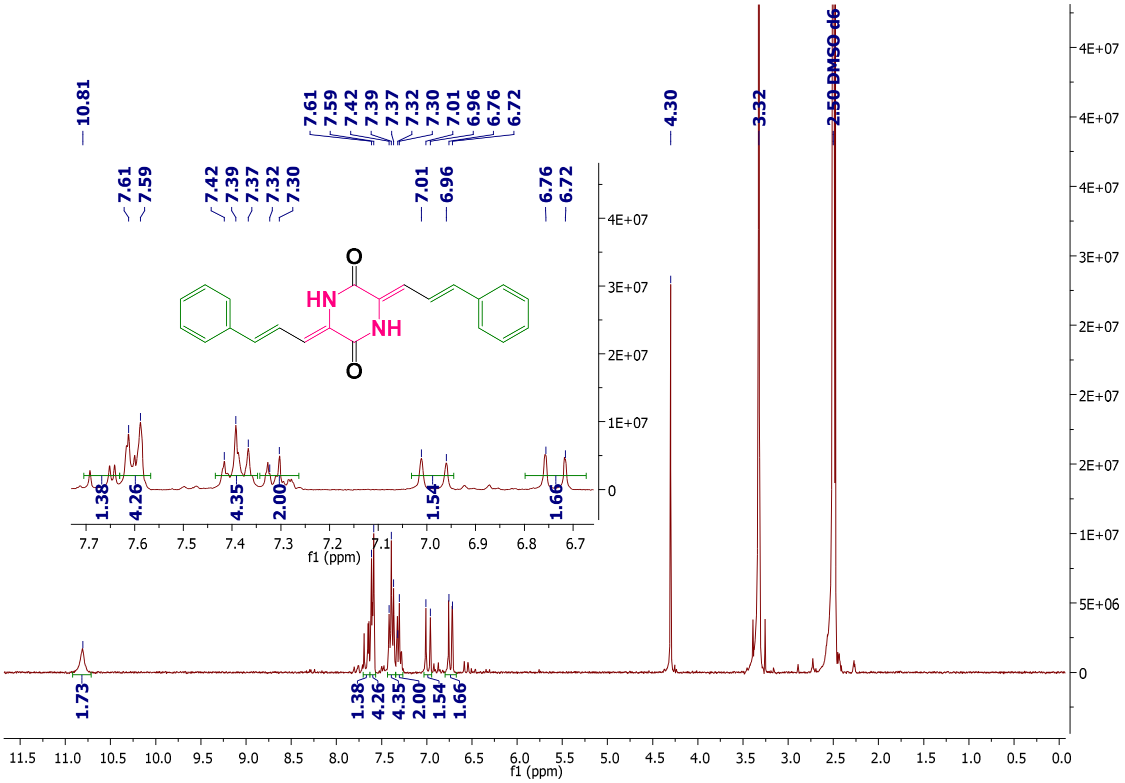

**Figure S16**: $^{1}$H NMR spectrum of **2c** (DMSO $d_6$, 300 MHz)

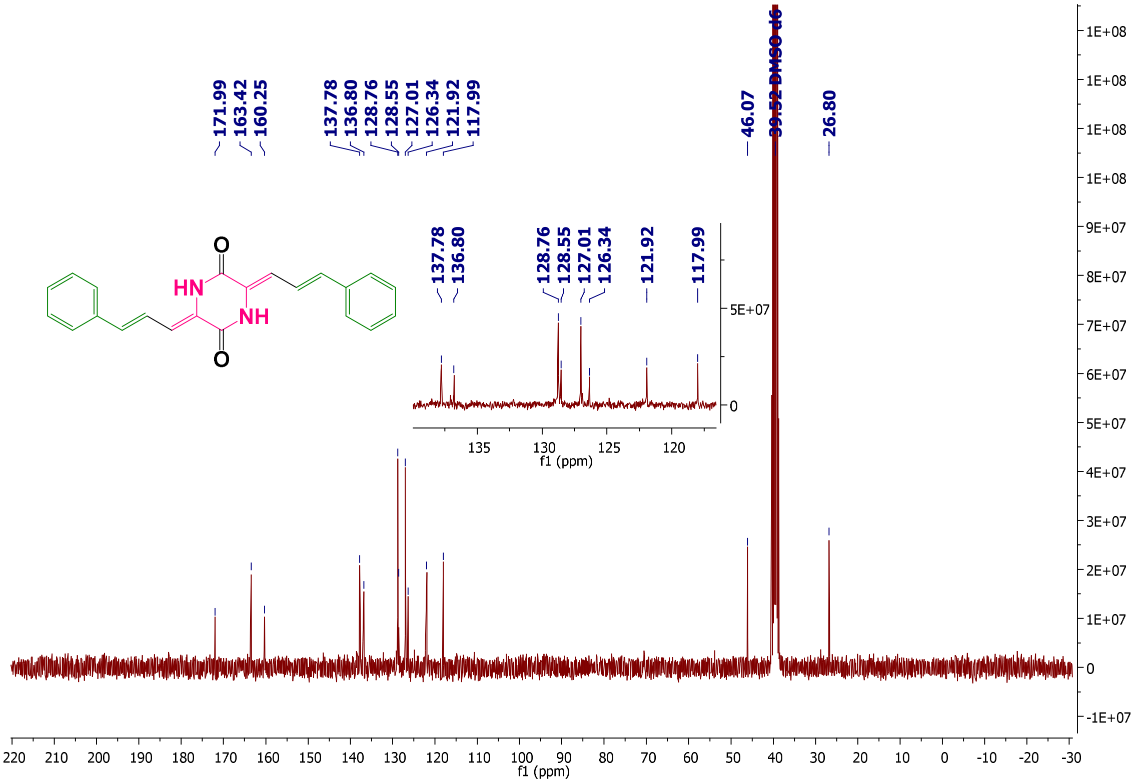

**Figure S17**: $^{13}$C NMR spectrum of **2c** (DMSO $d_6$, 75 MHz)

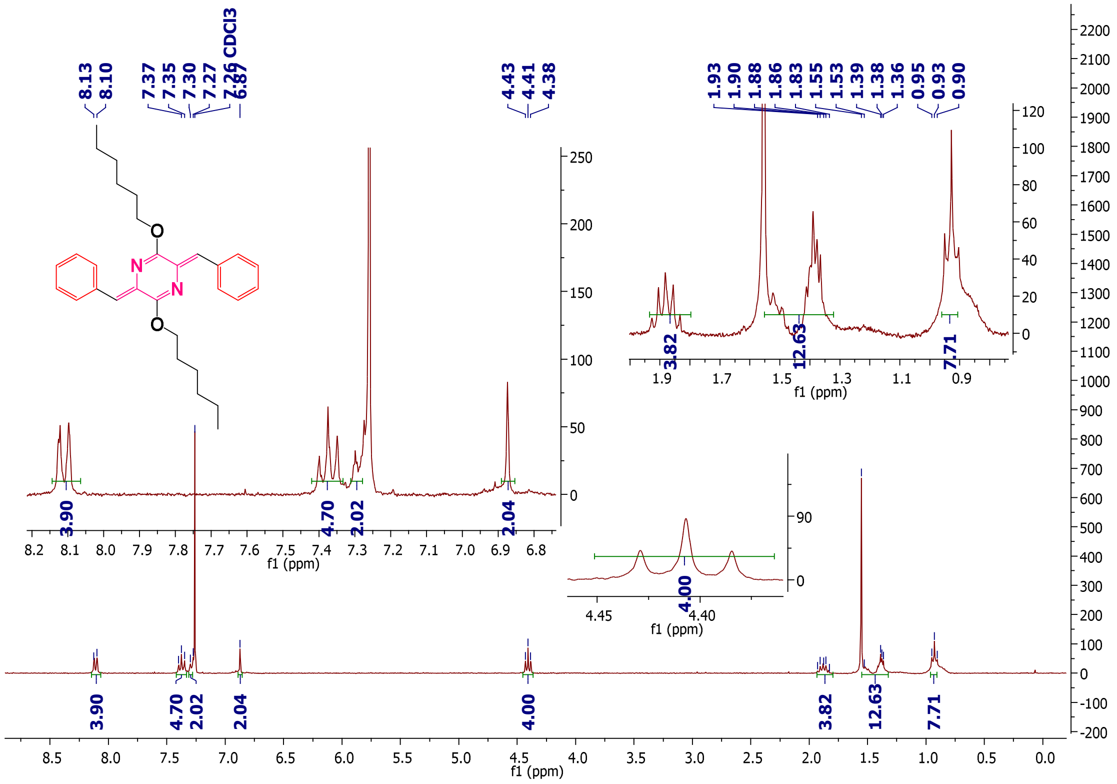

**Figure S18**: $^{1}H$ NMR spectrum of **AQM1** ($CDCl_3$, 300 MHz)

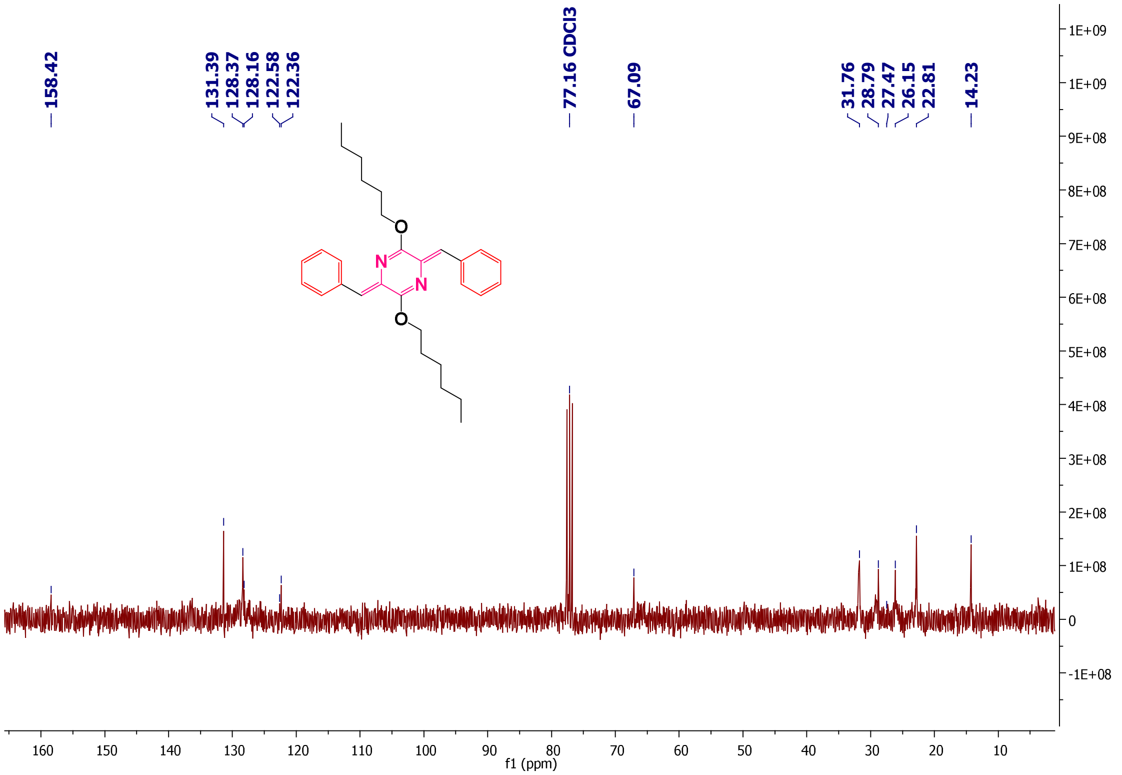

**Figure S19**: $^{13}C$ NMR spectrum of **AQM1** ($CDCl_3$, 75 MHz)

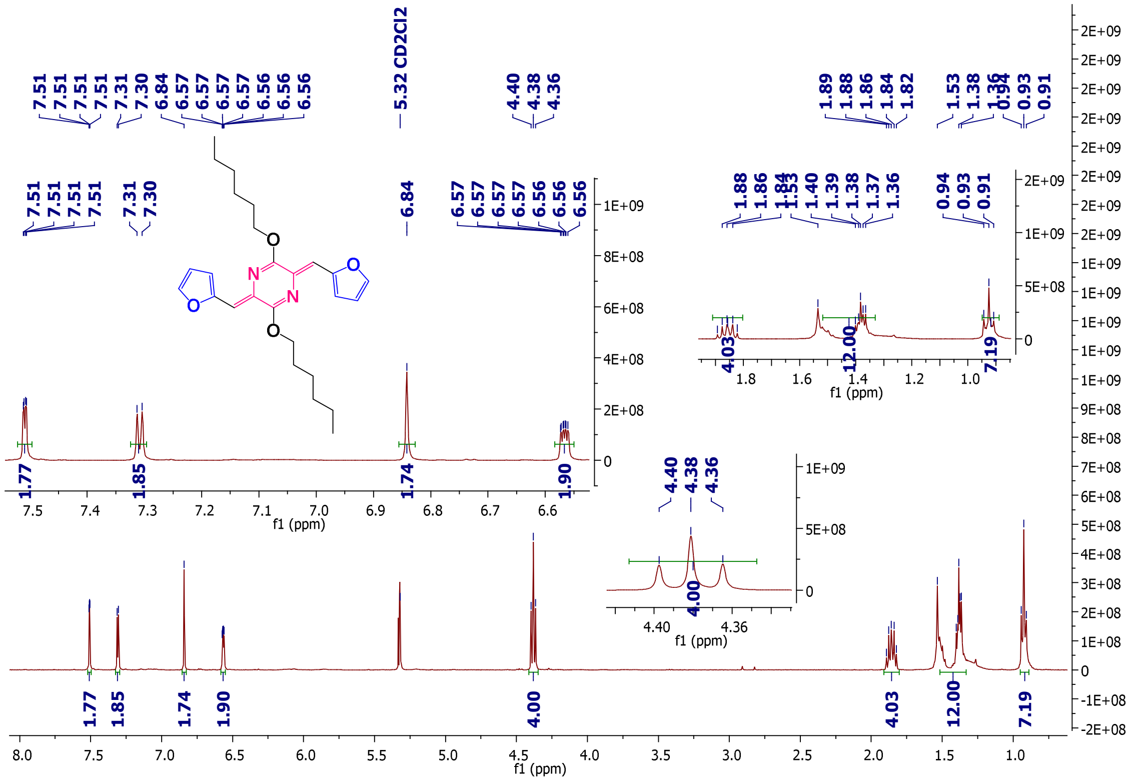

**Figure S20**: $^{1}$H NMR spectrum of **AQM2** ($CD_2Cl_2$, 400 MHz)

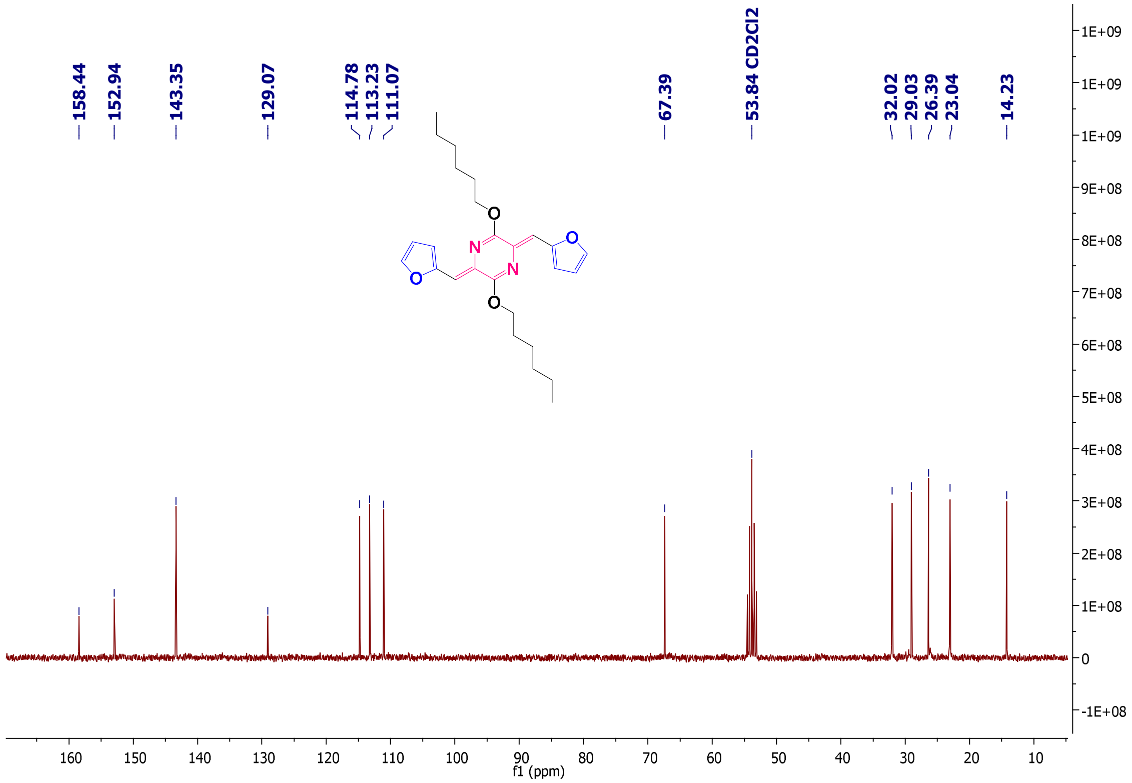

**Figure S21**: $^{13}$C NMR spectrum of **AQM2** ($CD_2Cl_2$, 75 MHz)

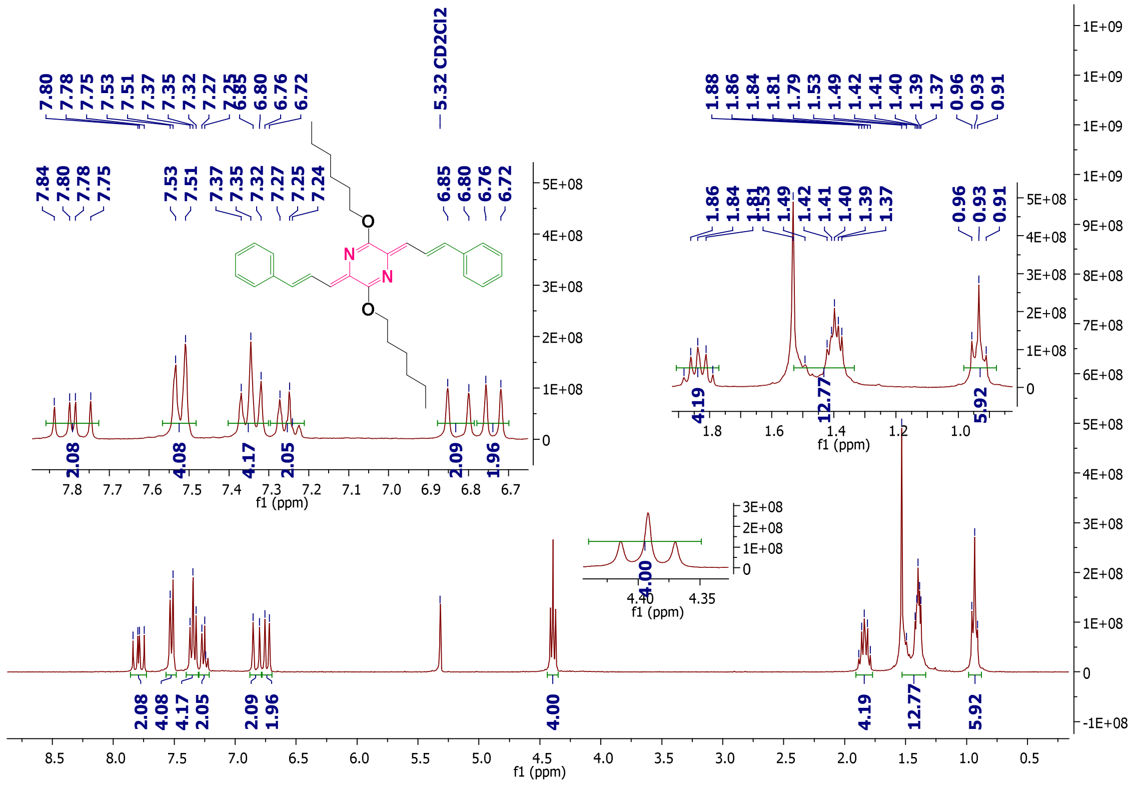


**Figure S22:** $^{1}$H NMR spectrum of **AQM3** ($CD_2Cl_2$, 300 MHz)

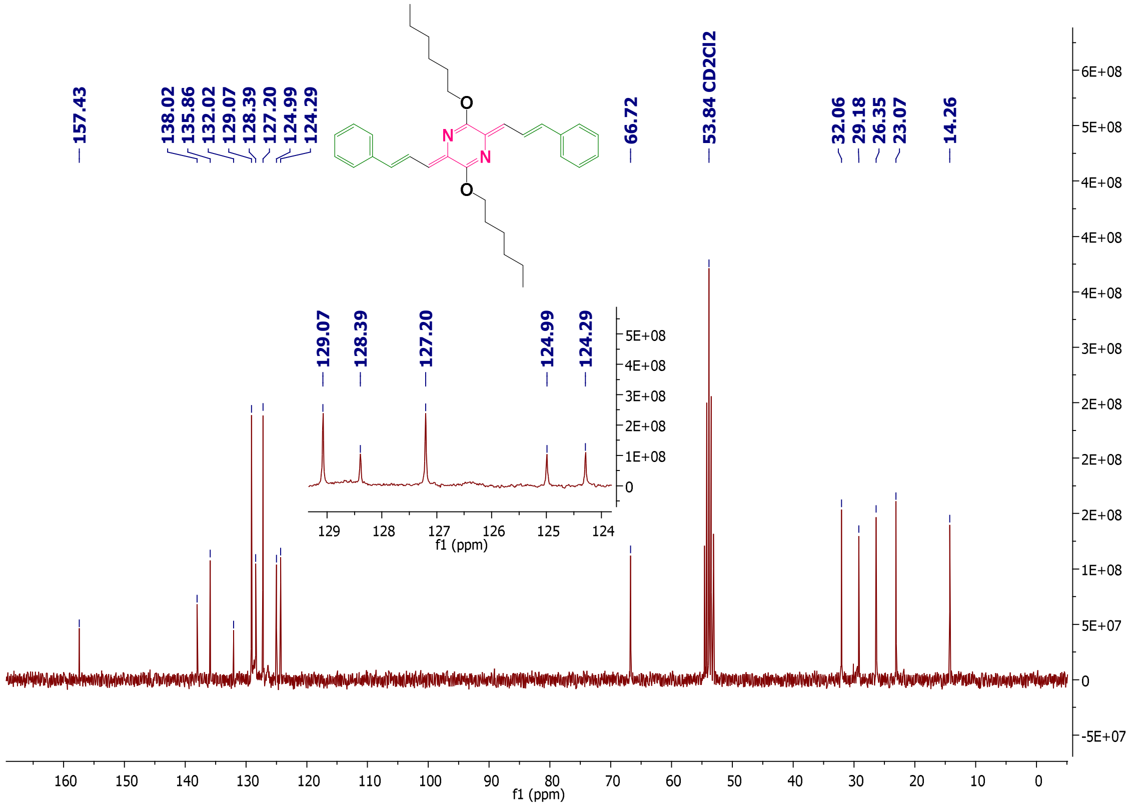


**Figure S23**: $^{13}$C NMR spectrum of **AQM3** ($CD_2Cl_2$, 75 MHz)

**References**

(1) Reiss, H.; Heller, A. The Absolute Potential of the Standard Hydrogen Electrode: A New Estimate. *J. Phys. Chem.* **1985**, *89* (20), 4207–4213. https://doi.org/10.1021/j100266a013.
